# Supplementary material for: Proof of crystal-field-perturbation-enhanced luminescence of lanthanide-doped nanocrystals through interstitial H+ doping
Source: Nat Commun. 2023 Sep 21;14:5870. doi: 10.1038/s41467-023-41411-6 (PMC10514317; doi:10.1038/s41467-023-41411-6)
Supplement: Supplementary file 1 — Supplementary Information [file 41467_2023_41411_MOESM1_ESM.pdf]

Supplementary Information

Proof of Crystal-Field-Perturbation-Enhanced Luminescence of Lanthanide-Doped Nanocrystals through Interstitial H^+ Doping

Guowei Li *et al*.

Corresponding author: Maochun Hong, hmc@fjirsm.ac.cn;
Yongsheng Liu, liuysh@fjirsm.ac.cn;
Lian Chen, cl@fjirsm.ac.cn

Supplementary Methods

The introduction of protons (H^+) inevitably affects the charge distribution in a crystal, resulting in changes in the polarizability of the coordinated atoms around the emitter, which further affects the transition dipole moment. Therefore, this situation is suitable to be studied by dynamic coupling mechanisms. For convenience, we considered only the contributions from multipole (lanthanide: Ln)-induced dipole–ligand (L) interactions for the dynamic coupling mechanism[1,2]. The Newman and Balasubramanian intensity parameters (denoted here by $A_{tp}{}^{\lambda}$) are related to the average polarizability of the ligand ($\bar{\alpha}_{\mathrm{L}}$) as[1]

$$A_{tp}{}^{\lambda}(\text{dynamic}) = 7(-1)^{p}\{t(2t-1)\}^{1/2}\begin{pmatrix}3\lambda3\\000\end{pmatrix}\langle r^{\lambda}\rangle\sum_{\mathrm{L}}\bar{\alpha}_{\mathrm{L}}\,R_{\mathrm{L}}{}^{-(t+1)}C_{-p}^{t}(\mathrm{L}), \qquad (S1)$$

where $\lambda = 2, 4, 6$; $t = \lambda \pm 1$; and $p = 0, \pm1\ \ldots, \pm t$. For cases where all Ln–L pairwise interactions are cylindrically symmetric (such as $[LnF_6]^{3-}$ octahedra), $A_{tp}{}^{\lambda}$ can be related to Axe's empirical $A_{tp}\Xi(t,\lambda)$ parameters on a one-to-one basis as

$$A_{tp}{}^{\lambda} = -A_{tp}\Xi(t,\lambda)\frac{2\lambda+1}{(2t+1)^{1/2}}. \qquad (S2)$$

According to Judd–Ofelt theory[3,4], the relationship between the oscillator strength parameter Ω_{λ} and $A_{tp}\Xi(t,\lambda)$ is expressed as

$$\Omega_{\lambda} = [\lambda][t]^{-1}\sum_{p,t}\left|A_{tp}\right|^{2}\Xi^{2}(t,\lambda). \qquad (S3)$$

Therefore, Ω_{λ} can be considered the sum of squares of $A_{tp}{}^{\lambda}$[1]. According to the relationship between the electric dipole oscillator strength f_{ed} and Ω_{λ}[5,6],

$$f_{\mathrm{ed}} = \frac{8\pi^{2}mcv}{3h(2J+1)}\chi_{\mathrm{ed}}\sum_{\lambda=2,4,6}\Omega_{\lambda}\langle 4f^{N}\psi J\left\|U^{\lambda}\right\|4f^{N}\psi' J'\rangle^{2}. \qquad (S4)$$

The relationship between the electric dipole transition probability A_{ed} and f_{ed} is expressed as

$$A_{\mathrm{ed}} = \frac{8\pi^{2}e^{2}v^{2}n^{2}}{mc}f_{\mathrm{ed}}, \qquad (S5)$$

where m, c, v, h, e, and n are the electron mass, speed of light in vacuum, transition wavenumber, Planck constant electron charge, and crystal refractive index, respectively. The A_{ed} values were obtained in this manner. Therefore, interstitial H^+ ions enhance the radiation fluorescence intensity based on their effect on the polarizability of the ligand, which can also be described as imposing an additional field around the emitter. This promotes the mixing of opposite parity configurations of lanthanide ions, ultimately leading to a change in A_{ed}.

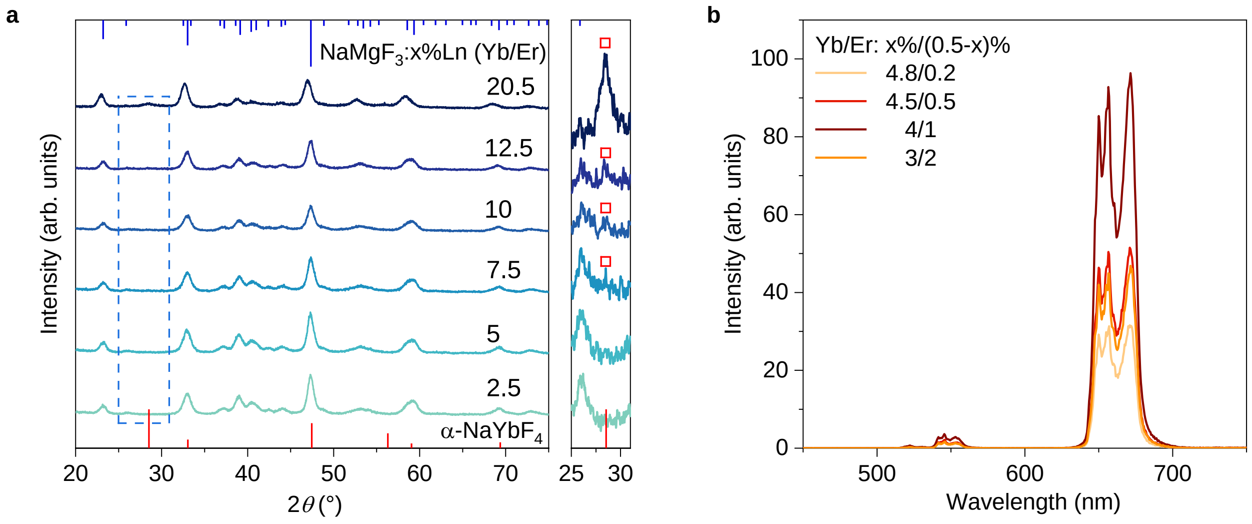

Supplementary Fig. 1 | Optimal rare earth doping ratio of $NaMgF_3$ NCs. a, Powder XRD patterns of as-synthesized $NaMgF_3$:Ln (Yb/Er) nanocrystals (NCs) with different rare earth doping concentrations (Ln^{3+} = 2.5–20.5 mol%). The right panel of **a** shows an enlarged view of the angular region from 25° to 31° with square boxes marking diffraction peaks corresponding to cubic $NaYbF_4$. **b**, UCL spectra for a series of Yb/Er co-doped $NaMgF_3$ NCs with different Yb/Er ratios. All UCL spectra were measured under identical experimental conditions using a 980 nm diode laser with a power density of 50 W/cm^2. Source data are provided as a Source Data file.

As shown in Supplementary Fig. 1a, when the nominal Ln^{3+} addition exceeds 5 mol%, diffraction peaks of cubic α-$NaYbF_4$ (JCPDS No. 77-2043) begin to appear in the XRD pattern (Supplementary Fig. 1a, right), in addition to the peaks of $NaMgF_3$:Ln. This occurs because the mismatch in ionic radius and valence state between Ln^{3+} and Mg^{2+} causes difficulty in effectively doping Yb^{3+} into the $NaMgF_3$ NCs[7,8], leading to the spontaneous formation of the α-$NaYbF_4$ phase by free Yb^{3+}. Therefore, to maintain the pure orthogonal phase of $NaMgF_3$:Ln, the nominal Ln^{3+} addition was maintained at 5 mol%. In addition, to obtain the highest UCL intensity, the ratio of Yb^{3+} to Er^{3+} was adjusted (Supplementary Fig. 1b). From this, the optimal Yb^{3+}/Er^{3+} doping ratio was determined to be 4/1 mol%.

Supplementary Table 1. Crystallographic data of orthorhombic-phase $NaMgF_3$ crystals (ICSD 94085).

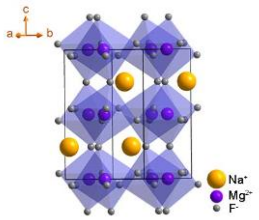

Atomic parameters of $NaMgF_3$ crystal						
Phase	Atom	Site	*x*/*a*	*y*/*b*	*z*/*c*	Symmetry
Orthorhombic	Na	4c	0.9896	0.0452	0.2500	C_s
	Mg	4b	0	0.5000	0	S_6
	F1	4c	0.0872	0.4741	0.2500	C_1
	F2	8d	0.7030	0.2953	0.0464	C_1

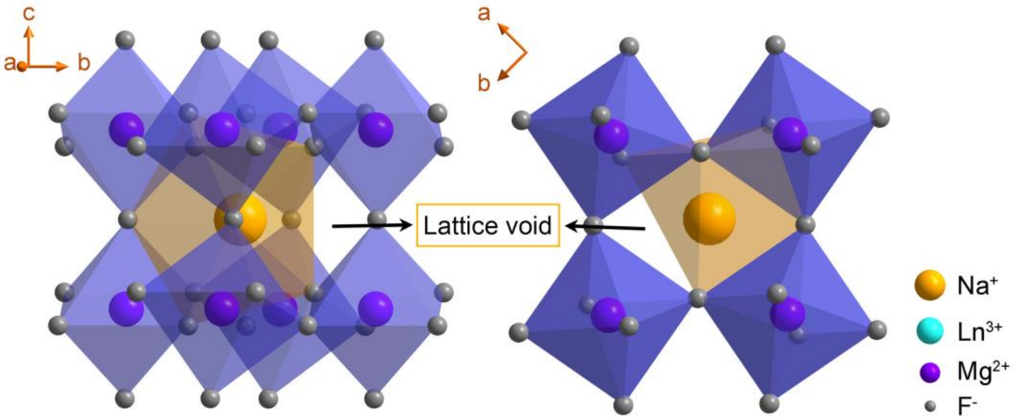

Supplementary Fig. 2 | Crystal structure of $NaMgF_3$. Schematic diagram of the sublattice structure of $NaMgF_3$. Na^+ ions fill dodecahedral cavities composed of eight $[MgF_6]^{4-}$ ortho-octahedra. However, Na^+ is smaller than the dodecahedral cavity and coordinates with the neighboring eight F^- anions to form distorted $[NaF_8]^{7-}$ dodecahedra. The four F^- anions at the distal sites do not contribute to the coordination of Na^+, resulting in the formation of a cavity structure (lattice void) by the four distal F^- anions. This cavity structure facilitates interstitial doping.

$NaMgF_3$ has an orthorhombic perovskite structure with space group *Pbnm* (Supplementary Table 1). The trivalent magnesium ions (Mg^{3+}) form ortho-octahedral structures ($[MgF_6]^{4-}$) with six surrounding fluorine anions (F^-) and have S_6 symmetry, with all Mg–F bond lengths measuring 2.01 Å[7,9]. The sodium ions (Na^+) fill tetrakaidekahedral cavities consisting of eight $[MgF_6]^{4-}$ octahedra, forming distorted $[NaF_8]^{7-}$ dodecahedra with eight surrounding F^- anions. This is because Na^+ ions are smaller than required to fill the tetrakaidekahedral cavities, causing the surrounding $[MgF_6]^{4-}$ octahedra to tilt and rotate toward the Na^+ ions to maintain the stability of the crystal structure. The neighboring $[MgF_6]^{4-}$ octahedra and $[NaF_8]^{7-}$ dodecahedra create an abundance of lattice voids[9]. Hydrogen ions (H^+) have a small ionic radius (0.012 Å); therefore, they easily enter the lattice voids to achieve crystal-field perturbation.

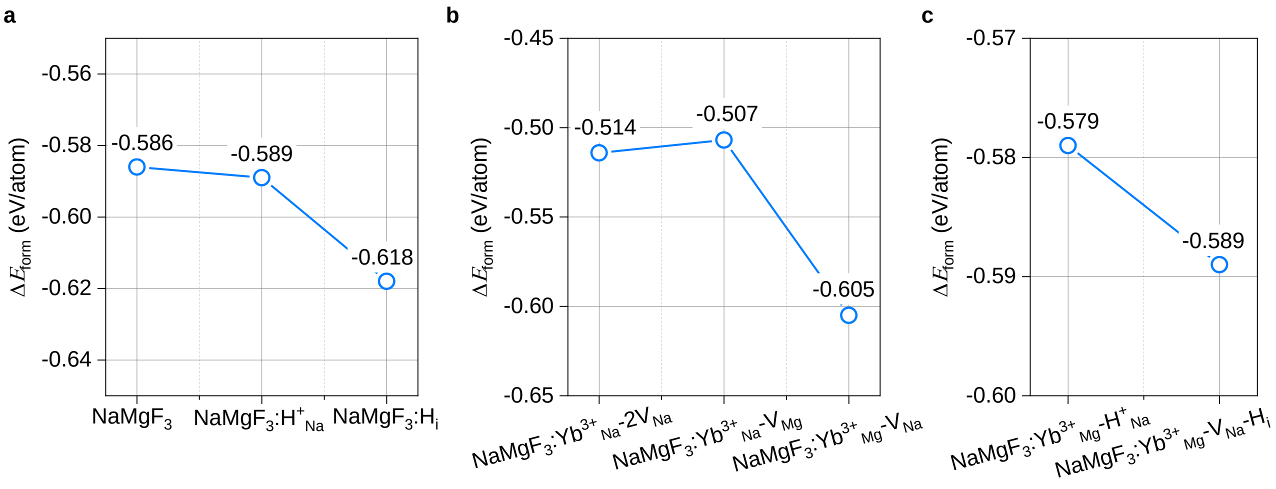

Supplementary Fig. 3 | Formation energy of $NaMgF_3$ NCs with different dopants. Formation energy (ΔE_{form}) per atom for **a**, $NaMgF_3$:H, **b**, $NaMgF_3$:Yb, and **c**, $NaMgF_3$:Yb/H crystals, as determined by DFT calculations. H^{+}_{Na} represents H^+ in a Na^+ site, Yb^{3+}_{Na} represents Yb^{3+} in a Na^+ site, Yb^{3+}_{Mg} represents Yb^{3+} in a Mg^{2+} site, V_{Na} is a Na^+ vacancy, and H_i is interstitial H^+. The results show that, for $NaMgF_3$ and $NaMgF_3$:Yb, interstitial H^+ doping ($NaMgF_3$:H_i in **a** and $NaMgF_3$:Yb^{3+}_{Mg}-V_{Na}-H_i in **c**) has a lower formation energy than other doping behaviors. In addition, Yb will preferentially occupy the Mg lattice sites ($NaMgF_3$:Yb^{3+}_{Mg}-V_{Na} in **b**) rather than the Na ones ($NaMgF_3$:Yb^{3+}_{Na}-V_{Mg} in **b**).

DFT calculations showed that the crystal structure formed by interstitial H^+-doping was more stable in both pure $NaMgF_3$ NCs and Yb^{3+}-doped $NaMgF_3$:Yb NCs compared to that formed by substitutional H^+-doping, suggesting that H^+ ions tend to fill the interstitial sites in $NaMgF_3$ and $NaMgF_3$:Yb lattices. Notably, the per-atom formation energy (ΔE_{form}; Supplementary Fig. 3) required for interstitial H^+-doping in the pure $NaMgF_3$ lattice (−0.618 eV) was slightly lower than that in the $NaMgF_3$:Yb lattice that had already been doped with Yb^{3+} (−0.589 eV), indicating that H^+ is more easily interstitially doped in pure $NaMgF_3$.

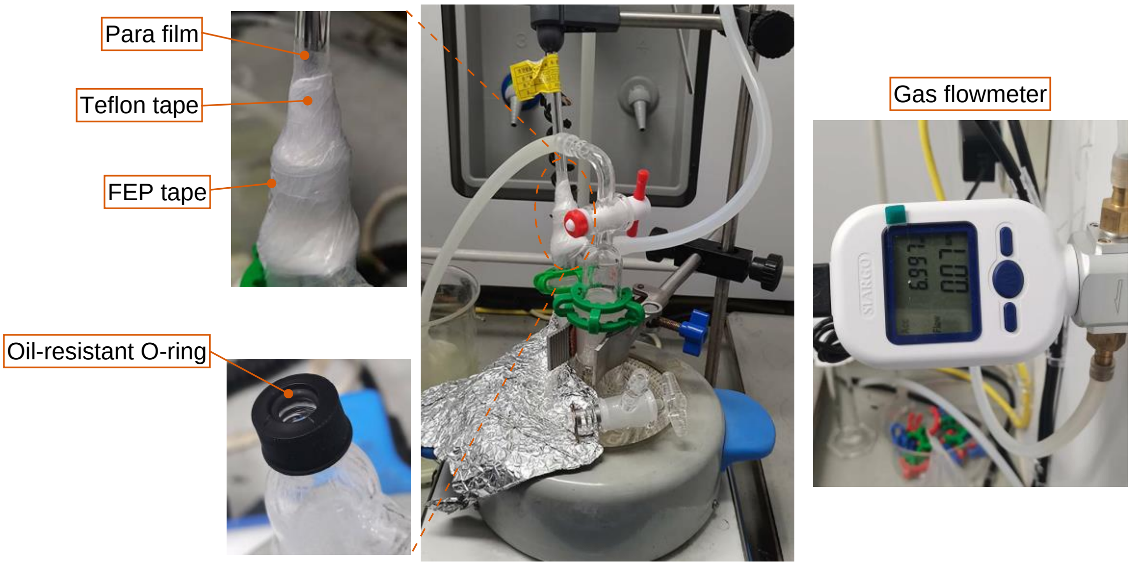

Supplementary Fig. 4 | Photographs of the reaction device. To prevent the leakage of overheated HAc vapor during the reaction process, it is crucial to completely seal the connection between the thermocouple (thermometer) and thermometer casing. Therefore, we sequentially wrapped the connection with parafilm, Teflon tape, and fluorinated ethylene propylene (FEP) tape. We also used a gas flowmeter to control the nitrogen (N_2) flow rate to prevent the HAc vapor from being blown out.

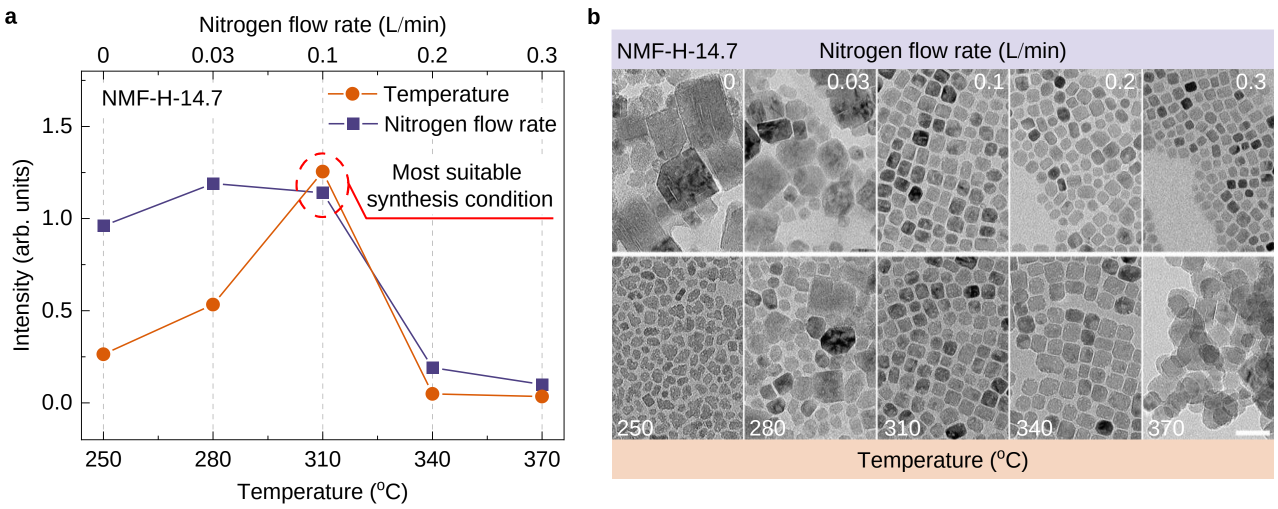

Supplementary Fig. 5 | Confirmation of the most suitable formation conditions of NMF-H-14.7 NCs. a, UCL intensity changes and **b**, corresponding transmission electron microscopy (TEM) images of NMF-H-14.7 NCs (H^+ source: HAc; Na^+ source: NaOH) synthesized at different nitrogen (N_2) flow rates and reaction temperatures. The experimental results showed that the strongest UCL intensity and best morphology of the NMF-H-14.7 NCs were obtained at a synthesis temperature of 310 °C and N_2 flow rate of 0.1 L/min. H^+ source: HAc; Na source: NaOH. Scale bars in **b**: 50 nm. Source data are provided as a Source Data file.

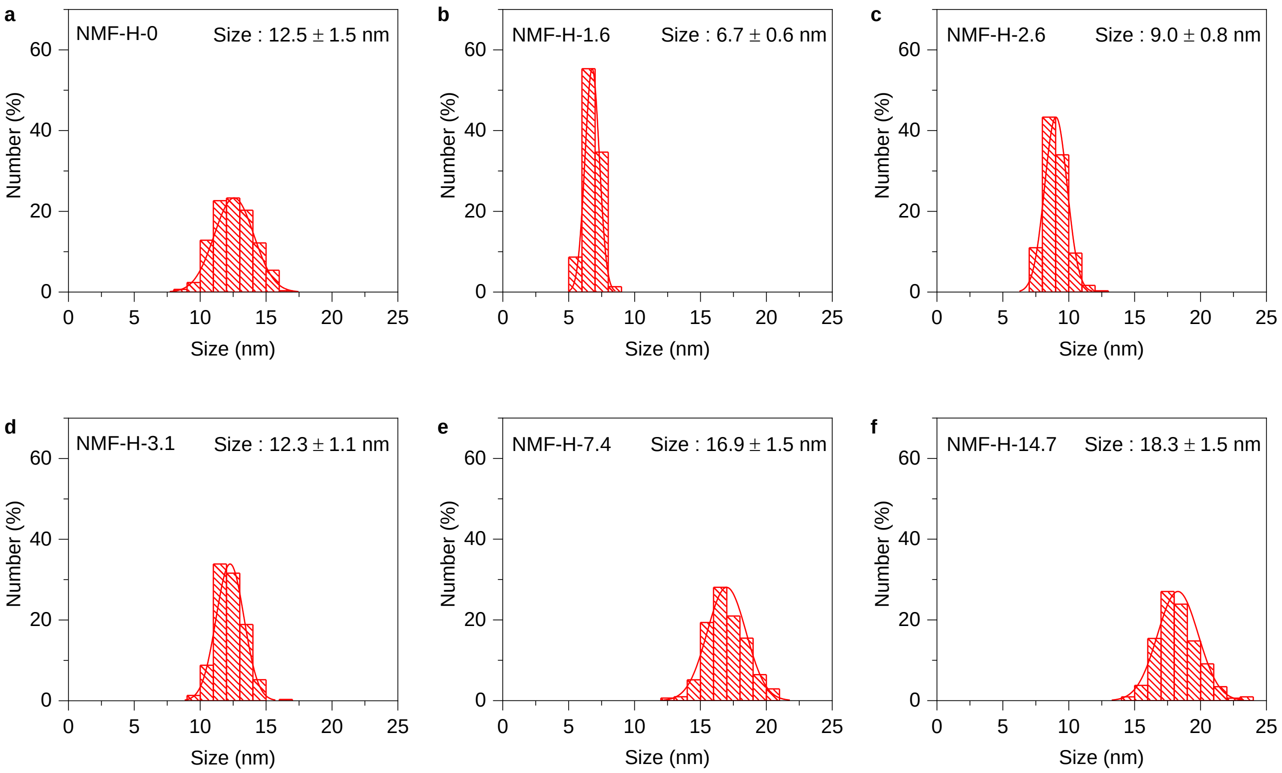

Supplementary Fig. 6 | Crystal size of $NaMgF_3$ NCs with different HAc precursor additions. Size distributions of as-synthesized **a**, NMF-H-0, **b**, NMF-H-1.6, **c**, NMF-H-2.6, **d**, NMF-H-3.1, **e**, NMF-H-7.4, and **f**, NMF-H-14.7 NCs (H^+ source: HAc; Na^+ source: NaOH). The results were obtained by measuring the sizes of 200 NCs in typical TEM images. The size shown in the upper right corner of each figure panel is the mean ± standard deviation. Source data are provided as a Source Data file.

The crystal size of the interstitially H^+-doped $NaMgF_3$ NCs first decreased and then increased with increasing interstitial H^+ content (Supplementary Fig. 6). This may be related to the variation of the formation energy (ΔE_{form}) of $NaMgF_3$:Ln^{3+} with the amount of interstitial H^+. DFT calculations showed that ΔE_{form} increases slightly with small amounts of interstitial H^+ doping, but further increases in the amount of interstitial H^+ doping result in a significant decrease in ΔE_{form} (Fig. 1c). Higher ΔE_{form} values mean that the corresponding substances are more difficult to form; therefore, in the same reaction time, NCs with a higher ΔE_{form} (e.g., NMF-H-1.6) form with a smaller particle size than those with a lower ΔE_{form} (e.g., NCs with increased amounts of interstitial H^+).

Supplementary Table 2. Results of refinement and calculated crystallographic data for NMF-H-X (X = 0, 1.6, 2.6, 3.1, 7.3 and 14.7 mmol HAc) NCs from Rietveld refinement of the high-resolution powder XRD patterns.

Parameter	Sample					
	NMF-H-0	NMF-H-1.6	NMF-H-2.6	NMF-H-3.1	NMF-H-7.3	NMF-H-14.7
a (Å)	5.491	5.497	5.498	5.495	5.487	5.487
b (Å)	7.677	7.682	7.668	7.68	7.669	7.668
c (Å)	5.357	5.359	5.369	5.361	5.361	5.361
α (°)	90	90	90	90	90	90
β (°)	90	90	90	90	90	90
γ (°)	90	90	90	90	90	90
V (Å^3)	225.823	226.285	226.313	226.216	225.597	225.579
Change ratio of V (%)	0	0.20	0.22	0.17	−0.10	−0.11
Size (Å)	104.6	85	90	114.7	265.8	290.2

Supplementary Table 3. Calculated structural parameters for $NaMgF_3$:Yb with different interstitial H^+ contents, as determined by first-principles DFT calculations.

Parameter	Sample defects			
	$NaMgF_3:Yb^{3+}_{Mg}$-V_{Na}	$NaMgF_3:Yb^{3+}_{Mg}$-V_{Na}-$1H_i$	$NaMgF_3:Yb^{3+}_{Mg}$-V_{Na}-$2H_i$	$NaMgF_3:Yb^{3+}_{Mg}$-V_{Na}-$3H_i$
Supercell	$Na_{15}Mg_{15}F_{48}Yb_1$	$Na_{15}Mg_{15}F_{48}Yb_1H_1$	$Na_{15}Mg_{15}F_{48}Yb_1H_2$	$Na_{15}Mg_{15}F_{48}Yb_1H_3$
H_i/(Mg+Yb)	0	6.3%	12.5%	18.8%
a (Å)	7.808	7.834	7.873	7.790
b (Å)	10.889	11.017	10.871	10.842
c (Å)	11.159	11.226	11.129	11.035
α (°)	90.142	90.323	89.703	90.053
β (°)	90.314	89.909	90.316	90.556
γ (°)	90.112	90.153	90.273	90.514
$\bar{R}_{Yb-F}$ (Å)	2.1149	2.1798	2.1115	2.1177
Supercell *V* ($Å^3$)	948.773	968.858	952.424	931.962
Unit-cell *V* ($Å^3$)[a]	237.193	242.215	238.106	232.991
Change ratio of *V* (%)[a]	0	2.12	0.38	−1.77

[a] The trend of variation obtained from first-principles calculations is similar to the results of high-resolution XRD fitting.

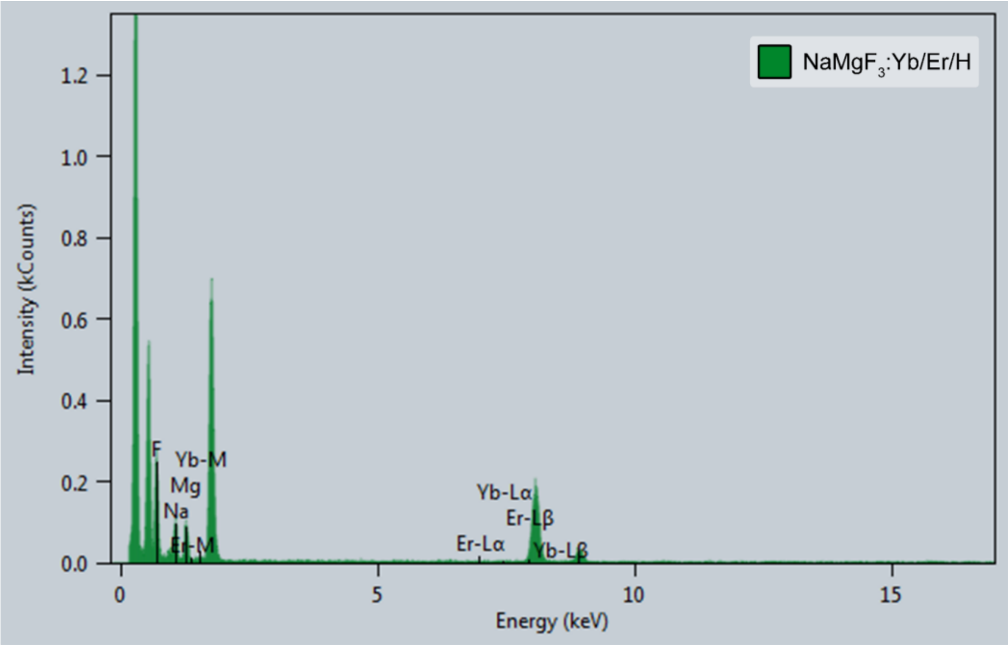

Supplementary Fig. 7 | Energy-dispersive X-ray spectrum of randomly selected NMF-H-3.1 NCs, showing the presence of host Na, Mg, and F ions and dopant Yb and Er ions. The spectrum confirms the successful co-doping of Yb^{3+} and Er^{3+} ions in the interstitially H^+-doped $NaMgF_3$:Yb/Er NCs.

Supplementary Table 4. Actual Yb^{3+} and Er^{3+} doping concentrations determined from ICP-OES spectrometry for $NaMgF_3$:Yb/Er interstitial H^+-doping using HAc as an H^+ precursor (nominal amounts ranging from 0 to 14.7 mmol HAc).

HAc (mmol)	Nominal Mg/Yb/Er contents (mol%)	Actual Mg/Yb/Er contents from ICP-OES (mol%)
14.7	95/4/1	98.77/1.05/0.18
7.3	95/4/1	98.69/1.11/0.19
3.1	95/4/1	98.37/1.40/0.23
2.6	95/4/1	98.15/1.58/0.27
1.6	95/4/1	97.92/1.77/0.30
0	95/4/1	98.64/1.16/0.20

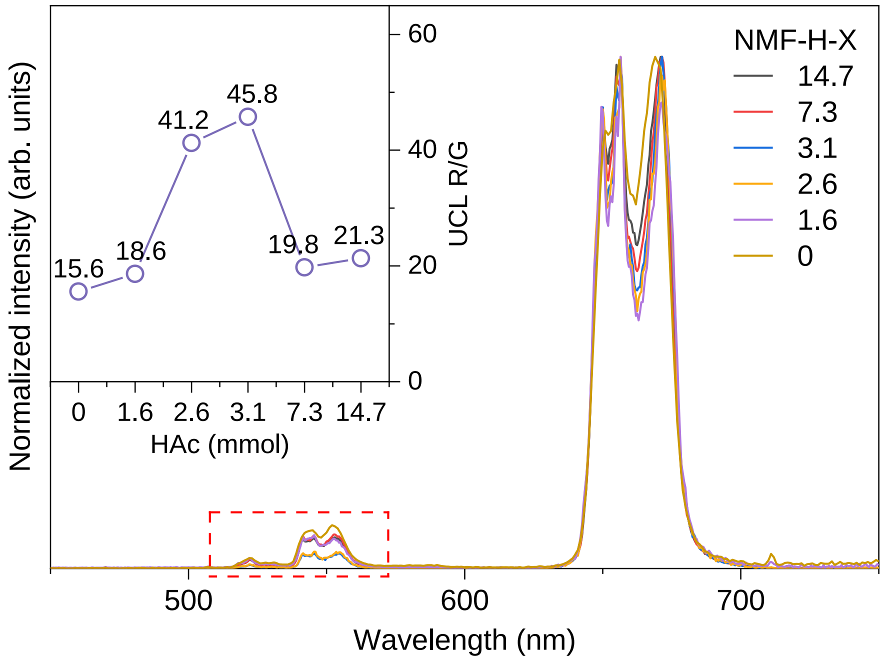

Supplementary Fig. 8 | The change of red-to-green (R/G) UCL intensity ratio with the addition of HAc. Normalized emission spectra of $NaMgF_3$:Yb/Er NCs as a function of the nominal amount of HAc (0–14.7 mmol). (Na^+ source: NaOH.) The inset displays the corresponding red-to-green (R/G) UCL intensity ratio. Red UCL wavelength: 615–725 nm, green UCL wavelength: 505–575 nm. As the nominal amount of HAc increases, the $NaMgF_3$:Yb/Er NCs maintain a high R/G UCL intensity ratio. NMF-H-2.6 and NMF-H-2.6 have higher R/G ratios than the other samples, because small concentrations of interstitial H^+ ions may slightly improve the symmetry of the $[LnF_6]^{3-}$ sublattice structure. Once the interstitial H^+ concentration increases past a certain point, the symmetry of the $[LnF_6]^{3-}$ octahedra will be weakened again (Supplementary Fig. 25). Overall, the interstitial H^+ ions only have a limited effect on the $[LnF_6]^{3-}$ sublattice structure (crystal-field perturbation); therefore, there is little change in the green UCL intensity in the normalized UCL spectra. Source data are provided as a Source Data file.

Supplementary Table 5. Upconversion- and NIR-quantum yield (UCQY and NIR-QY, respectively) of NMF-H-X (X = 0, 1.6, 2.6, 3.1, 7.3, and 14.7 mmol HAc) NCs.

Parameter	Sample					
	NMF-H-0[a]	NMF-H-1.6[a]	NMF-H-2.6	NMF-H-3.1	NMF-H-7.3	NMF-H-14.7
UCQY (%)	<0.01	<0.01	0.029	0.036	0.165	0.180
NIR-QY (%)	<0.01	<0.01	8.23	8.99	15.87	20.87

[a]The UCQY and NIR-QY data for NMF-H-0 and NMF-H-1.6 were below the detection limit of the instrument (<0.01%).

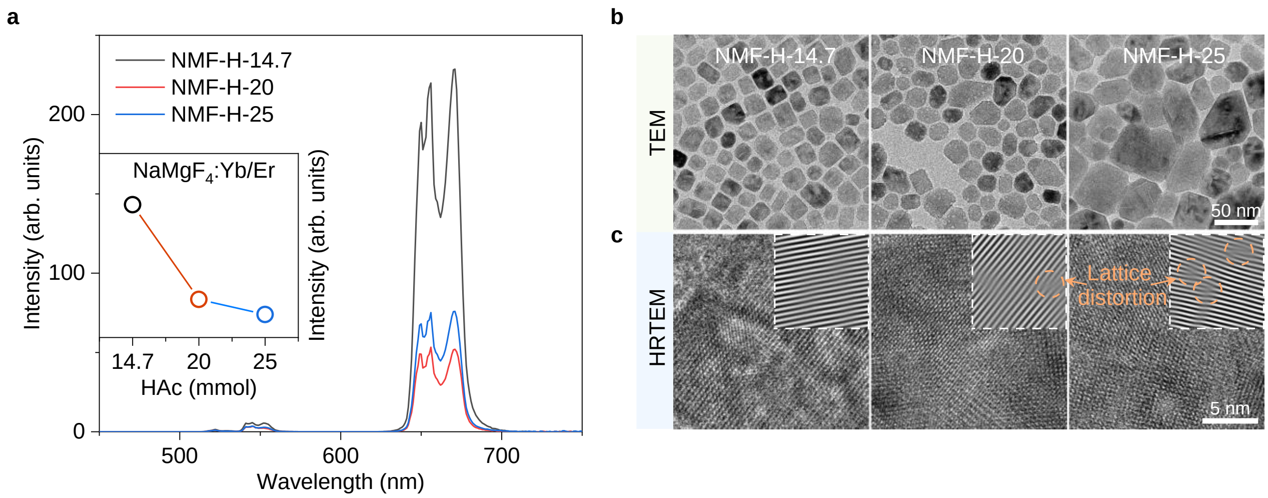

Supplementary Fig. 9 | Lattice distortion at high interstitial H^+-doping content. a, Typical UCL spectra, **b**, TEM images, and **c**, corresponding high-resolution TEM (HRTEM) images of as-synthesized $NaMgF_3$:Yb/Er NCs with excess HAc added during the synthetic procedure (14.7–25 mmol) (Na^+ source: NaOH). The inset in **a** shows the UCL intensity as a function of HAc content. The TEM images in **b** show that excessive interstitial H^+ doping alters the $NaMgF_3$:Yb/Er NC morphology. The Fourier-filtered HRTEM images in the insets in **c** reveal that excessive interstitial H^+ doping leads to significant lattice distortion, which may be the main reason for the decrease of crystal fluorescence intensity. Source data are provided as a Source Data file.

The luminescence intensity of $NaMgF_3$:Yb/Er NCs reached a maximum when the nominal amount of HAc was 14.7 mmol, and further increases in the HAc addition led to a decrease in the luminescence intensity and change of crystal morphology (Supplementary Fig. 9). Because interstitial H^+ ions are essentially interstitial atomic defects, large amounts of H^+ doping led to lattice distortion (e.g., changes in the lattice fringes, as shown in Supplementary Fig. 9c). Severe lattice distortion can cause fluorescence quenching and a reduction in luminescence intensity. Therefore, only a small amount of HAc (≤14.7 mmol) was added to the reaction solvent to illustrate the crystal field perturbation effect.

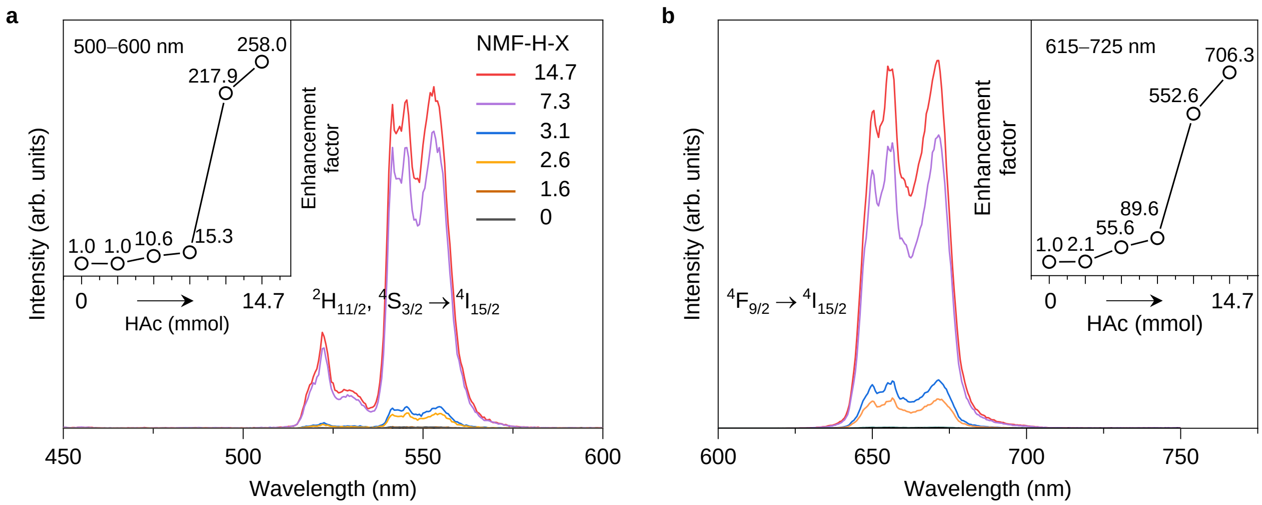

Supplementary Fig. 10 | a, Green and **b**, red UCL spectra (450–600 nm) of $NaMgF_3$:Yb/Er NCs synthesized with HAc (nominal amounts of 0–14.7 mmol) (Na^+ source: NaOH) under 980 nm diode laser excitation with a power density of 50 W/cm^2. The insets in **a** and **b** show the corresponding enhancement factors of the green and red UCL intensities for NMF-H-X (with HAc). The interstitial H^+ doping strategy effectively enhanced the green and red UCL intensities simultaneously, with enhancement factors of up to 258 and 706.3 for green ($^2H_{11/2}$,$^4S_{3/2}$ → $^4I_{15/2}$) and red ($^4F_{9/2}$ → $^4I_{15/2}$) emissions, respectively. Source data are provided as a Source Data file.

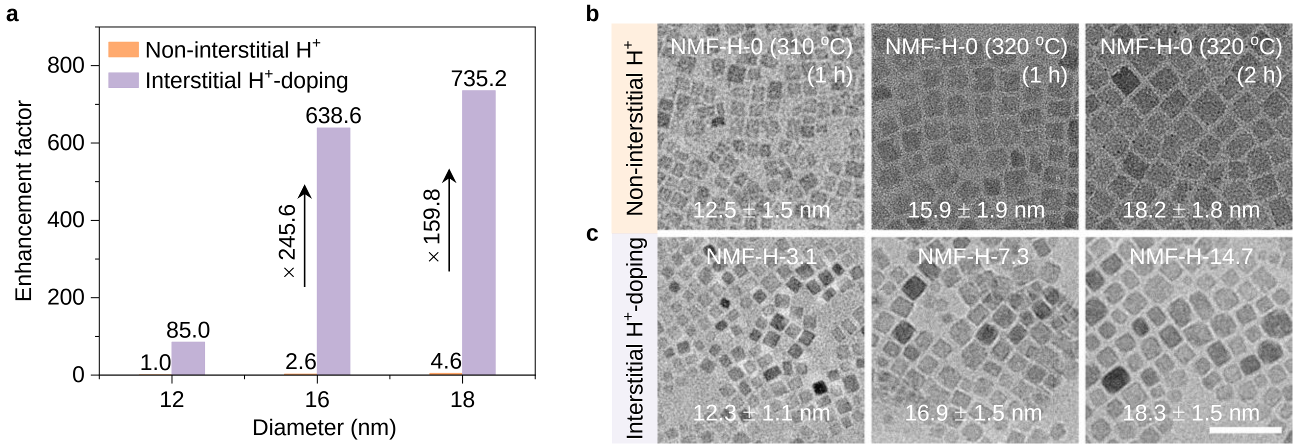

Supplementary Fig. 11 | Effect of interstitial H^+ doping on UCL of $NaMgF_3$:Yb/Er NCs at the same size. a, UCL enhancement factors of interstitially H^+-doped $NaMgF_3$:Yb/Er NCs relative to NMF-H-0 (without H^+-doping) samples with a similar NC size (Na^+ source: NaOH). **b,** TEM images of non-interstitially H^+-doped $NaMgF_3$:Yb/Er NCs with different sizes (as listed at the bottom of each panel in **b**) synthesized at different reaction temperatures and times (as listed at the top of each panel in **b**). TEM images of interstitially H^+-doped $NaMgF_3$:Yb/Er NCs with different sizes (as listed at the bottom of each panel in **c**) as a function of the nominal amount of HAc used in the synthetic procedure (X = 3.1, 7.3, 14.7 mmol). Scale bars in **b** and **c**: 50 nm.

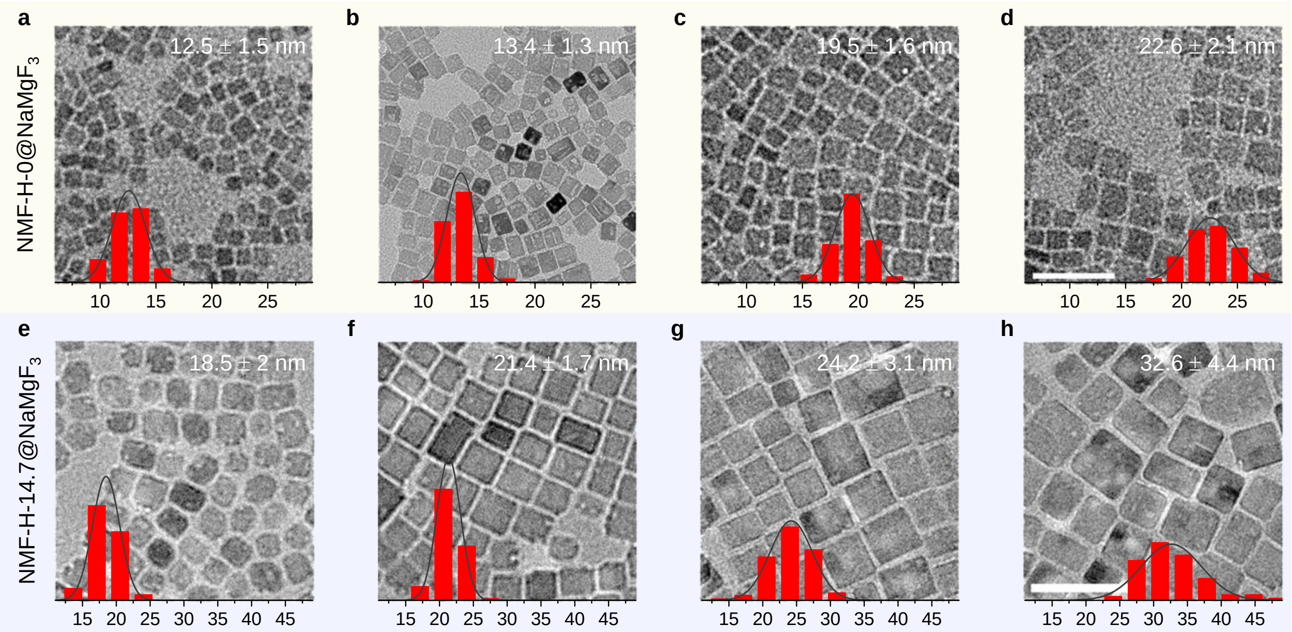

Supplementary Fig. 12 | TEM images and crystal size distribution of NMF-H-0@$NaMgF_3$ and NMF-H-14.7@$NaMgF_3$ core–shell NCs with inert $NaMgF_3$ shell thicknesses of **a–d**, 0–5.1 nm and **e–h**, 0–7.1 nm. The values in the upper right corner of each figure panel are the mean crystal size ± standard deviation obtained by measuring the sizes of about 150 NCs from typical TEM images. Scale bar: 50 nm. The gradual increase in NC size indicates that the NMF-H-0 and NMF-H-14.7 NCs were successfully coated with an inert $NaMgF_3$ shell. Source data are provided as a Source Data file.

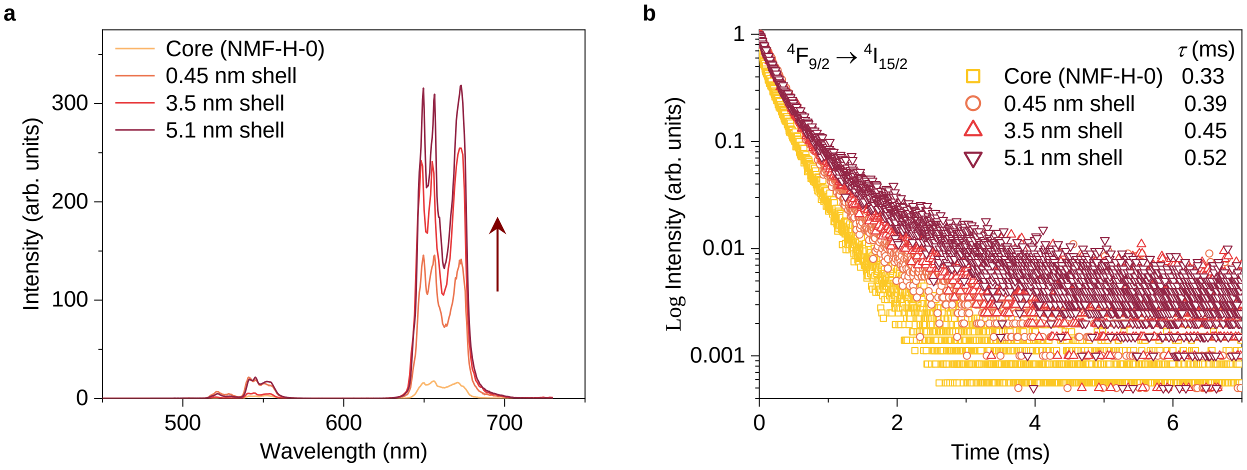

Supplementary Fig. 13 | Effect of inert $NaMgF_3$ shell on NMF-H-0 NCs. a, Typical UCL spectra and **b**, lifetimes of Er^{3+} emitters ($^4F_{9/2}{\rightarrow}^4I_{15/2}$) in NMF-H-0@$NaMgF_3$ core–shell NCs as a function of the thickness of the inert $NaMgF_3$ shell. Source data are provided as a Source Data file.

Similarly to conventional UCL NCs[10,11], the epitaxial growth of an $NaMgF_3$ inert shell on the surface of the NMF-H-0 NCs effectively reduced the influence of surface defects and the external environment on the fluorescence properties of NMF-H-0@$NaMgF_3$ core–shell NCs. The UCL intensity and lifetime of the NMF-H-0@$NaMgF_3$ NCs increased with increasing inert shell thickness (Supplementary Fig. 13).

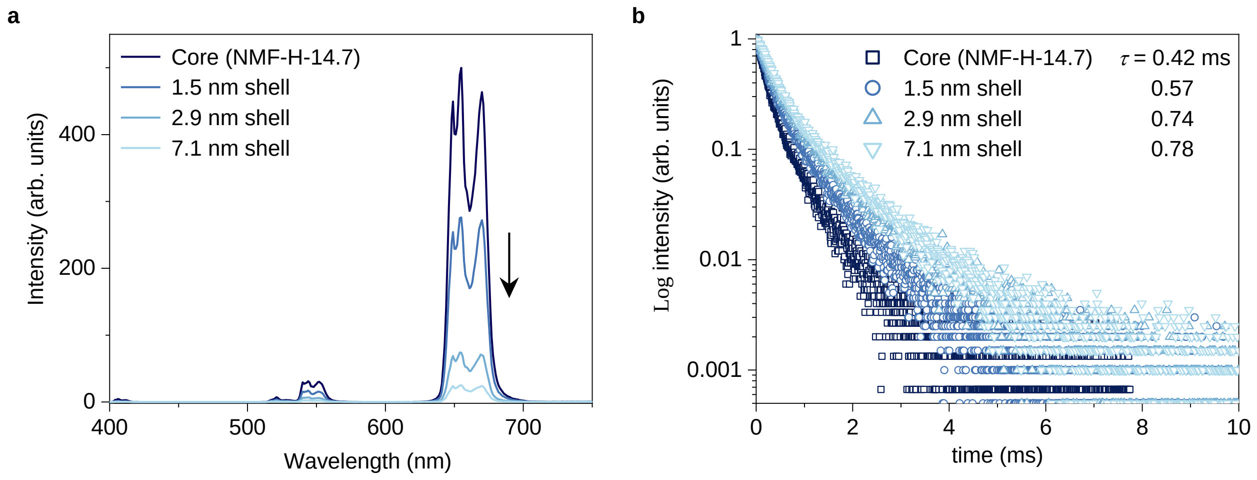

Supplementary Fig. 14 | Effect of inert $NaMgF_3$ shell on NMF-H-14.7 NCs. a, Typical UCL spectra and **b**, lifetimes of Er^{3+} emitters ($^4F_{9/2}\rightarrow{}^4I_{15/2}$) in NMF-H-14.7@$NaMgF_3$ core–shell NCs as a function of the thickness of the inert $NaMgF_3$ shell. Source data are provided as a Source Data file.

The UCL lifetimes of Er^{3+} emitters in the $^4F_{9/2}$ state for the NMF-H-14.7 NCs were gradually prolonged as the thickness of the inert $NaMgF_3$ shell increased (Supplementary Fig. 14b). This implies that the epitaxial coating of NMF-H-14.7 NCs by an inert $NaMgF_3$ shell was successful. In addition, the surface quenching effect was effectively mitigated. However, in contrast to conventional core–shell NCs, the UCL intensity of the NMF-H-14.7@$NaMgF_3$ NCs steadily diminished as the thickness of the inert $NaMgF_3$ shell increased (Supplementary Fig. 14a). The formation energy for interstitial H^+-doping is much lower in pure $NaMgF_3$ than in $NaMgF_3$:Yb/Er; therefore, we speculated that the H^+ ions diffused from the NMF-H-14.7 core to the $NaMgF_3$ inert shell, causing the crystal-field state of the NMF-H-14.7 core to gradually return to its original state.

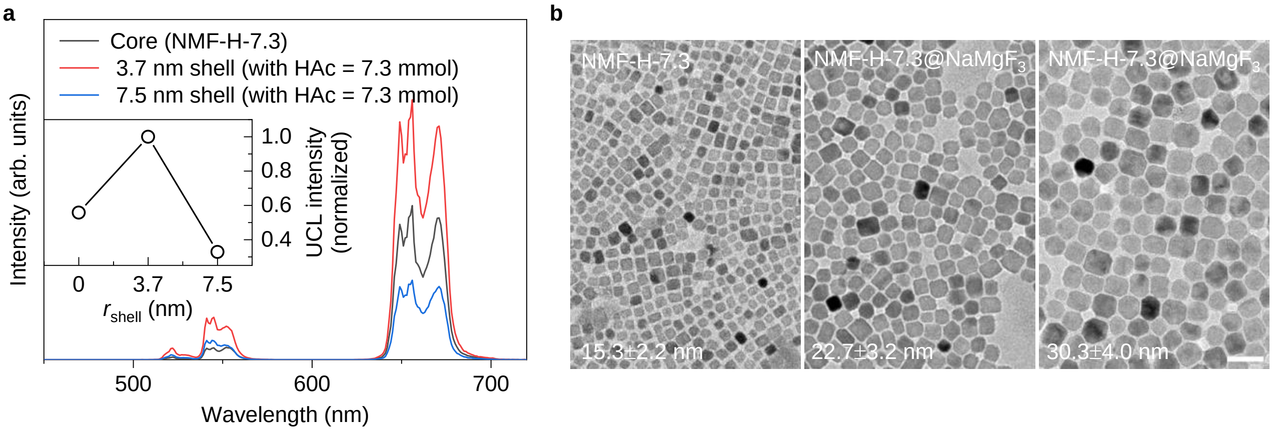

Supplementary Fig. 15 | Effect of interstitial H^+ doped $NaMgF_3$:H shell on NMF-H-7.3 NCs. a, Typical UCL spectra and **b**, TEM images of the NMF-H-7.3 and NMF-H-7.3@$NaMgF_3$:H core–shell NCs as a function of the thickness of the H^+-doped $NaMgF_3$ shell (scale bar: 50 nm). The inset in **a** shows the normalized changes in UCL intensity of NMF-H-7.3@$NaMgF_3$:H NCs with different shell thicknesses upon continuous 980 nm NIR diode laser excitation at a power density of ~50 W/cm^2. The values in the bottom left corner of each panel of **b** are the mean crystal size ± standard deviation obtained by measuring the size of 100 NCs from typical TEM images. Scale bars in **b**: 50 nm. Source data are provided as a Source Data file.

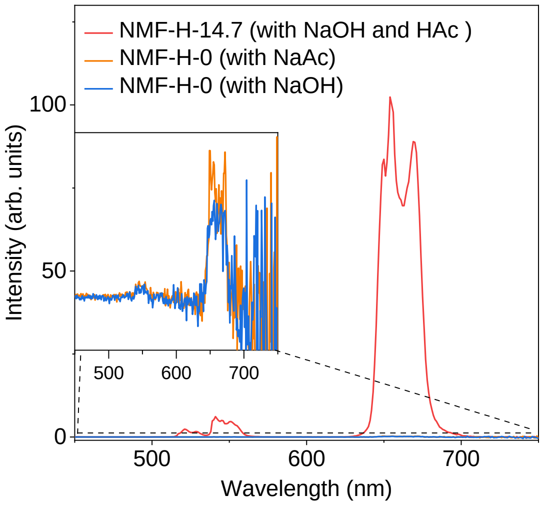

Supplementary Fig. 16 | Comparison of the typical UCL spectra of NMF-H-14.7 (with NaOH and HAc), NMF-H-0 (with NaAc), and NMF-H-0 (with NaOH) NCs under 980 nm diode laser excitation with a power density of 50 W/cm^2. Owing to the weak luminescence intensity of the NMF-H-0 samples without interstitial H^+ doping, the inset shows an enlarged view of the NMF-H-0 (with NaAc or NaOH) spectra below the dashed line. The UCL intensity of NMF-H-0 (with NaAc) was slightly stronger than that of NMF-H-0 (with NaOH), because NaOH inevitably introduced trace amounts of OH^- anions to the NCs, which quenched the luminescence of the Ln^{3+} ions to some extent. Source data are provided as a Source Data file.

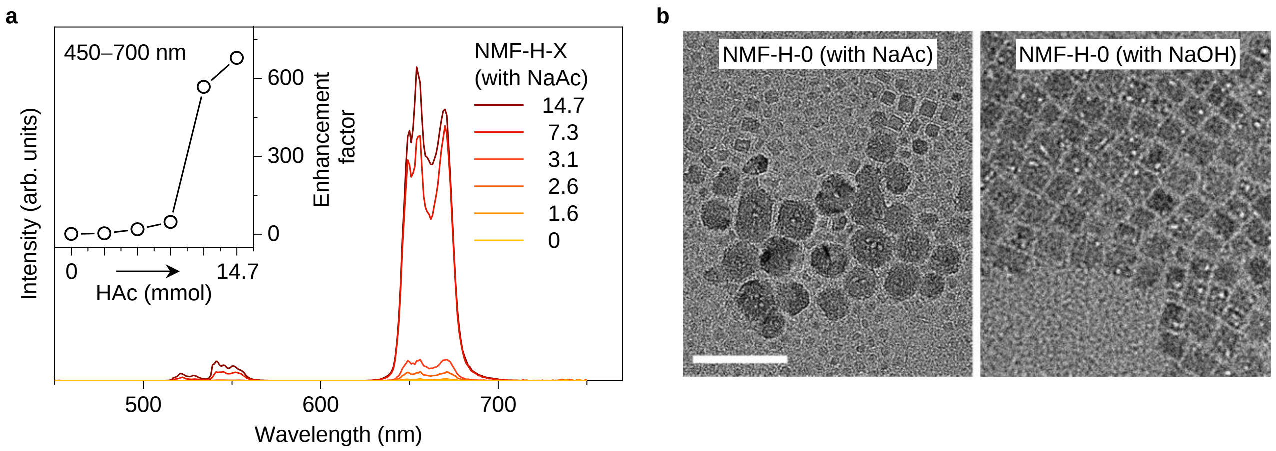

Supplementary Fig. 17 | Effect of NaAc on interstitial H^+ doping strategy. a, Typical UCL spectra of $NaMgF_3$:Yb/Er (with NaAc) NCs as a function of the nominal amount of HAc (X = 0–14.7 mmol) added during synthesis. The inset shows the corresponding UCL enhancement factors for NMF-H-X (with NaAc and HAc). The increasing UCL intensity with HAc content indicates that changing the Na^+ source does not affect the implementation of the interstitial H^+ doping strategy. **b**, TEM images of NMF-H-0 NCs synthesized with NaAc (left) and NaOH (right). Although the interstitial H^+ doping strategy can also be achieved using NaAc as the Na^+ source, good crystal morphology cannot be obtained. Scale bars in **b**: 50 nm. Source data are provided as a Source Data file.

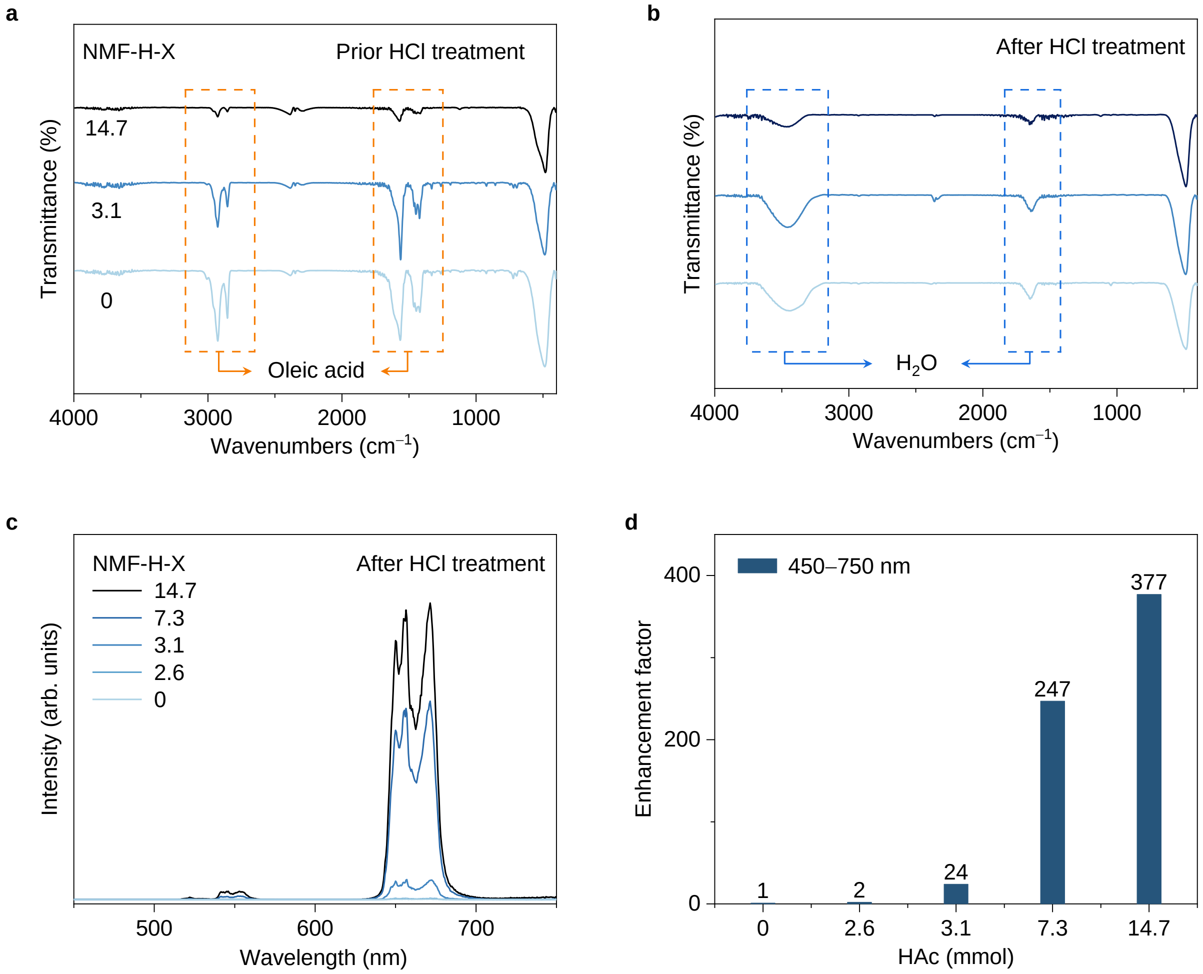

Supplementary Fig. 18 | Effect of surface ligands on interstitial H^+ doping strategy. a, Fourier-transform infrared (FTIR) spectra of NMF-H-0, NMF-H-3.1, and NMF-H-14.7 NCs (H^+ source: HAc; Na^+ source: NaOH). All three spectra contain characteristic absorption peaks of oleic acid at 2927.8 and 2856.4 cm^{-1} (stretching vibrations of –CH_2) and 1560.3 and 1438.8 cm^{-1} (stretching vibrations of –COO–), indicating that the NMF-H-0, NMF-H-3.1, and NMF-H-14.7 NCs all have similar crystal surface environments. **b**, FTIR spectra of NCs after acid washing. The characteristic absorption peaks of oleic acid disappear, but characteristic peaks of water molecules (at 3444.7 and 1637.5 cm^{-1}) are present, indicating that the oleic acid ligands are completely removed and replaced by a layer of adsorbed water molecules. **c**, UCL spectra of NMF-H-X (X = 0–14.7 mmol HAc) NCs after acid washing and **d**, corresponding UCL enhancement factors. After the complete removal of surface ligands, the emission intensity of the NMF-H-X NCs still increased as the interstitial H^+ content increased, indicating that changes in the surface ligands do not have a significant impact on the implementation of the interstitial H^+-doping strategy. Source data are provided as a Source Data file.

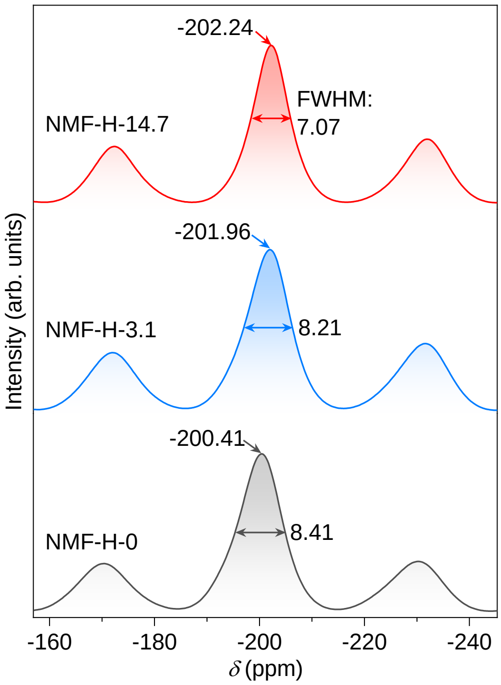

Supplementary Fig. 19 | ^{19}F SSNMR spectroscopy verified the successful interstitial H^+-doping. ^{19}F SSNMR spectra of NMF-H-0, NMF-H-3.1, and NMF-H-14.7 NCs (magic-angle spinning (MAS) frequency (ν): 14 kHz). The chemical shifts were externally referenced to benzotrifluoride (−63.7 ppm). Source data are provided as a Source Data file.

From the obtained ^{19}F SSNMR spectra (Supplementary Fig. 19), we noted that the spectral peak gradually shifted to the high frequency field with an increase in the nominal addition of HAc. Notably, NMF-H-0 and NMF-H-3.1 had analogous linewidths (FWHMs of NMF-H-0 and NMF-H-3.1 was 8.41 and 8.21 ppm, respectively) as well as identical particle sizes (~12 nm), indicating that they have similar surface chemical environments[12]. Therefore, the shift in the ^{19}F SSNMR spectral peak between the spectra of NMF-H-0 to NMF-H-3.1 (by 1.55 ppm from −200.41 to −201.96 ppm) means that the chemical environment around F atoms within the crystal changed[12]. Upon effective interstitial H^+ doping, H–F bonds form, and the strongly electronegative F^- ions lead to electron deflection toward F^-. This enhances the electron shielding effect on F^- and causes a chemical shift of the ^{19}F SSNMR spectra toward the high frequency field. When the nominal addition of HAc was increased further, it only caused a slight chemical shift. For example, for NMF-H-3.1 and NMF-H-14.7, a

chemical shift of only 0.28 ppm was observed, which implies that the content of interstitial H^+ defects in $NaMgF_3$:Yb/Er NCs had reached its limit. Furthermore, the linewidth significantly narrowed (FWHMs of NMF-H-0 and NMF-H-14.7 was 8.21 and 7.07 ppm, respectively) because of the increased crystal size. To correctly reflect the chemical environment changes around F^- ions in the NCs and reduce the influence of surface ligands in the experiments, the samples for ^{19}F SSNMR spectroscopy were acid-washed to a bare core state for testing (see Methods).

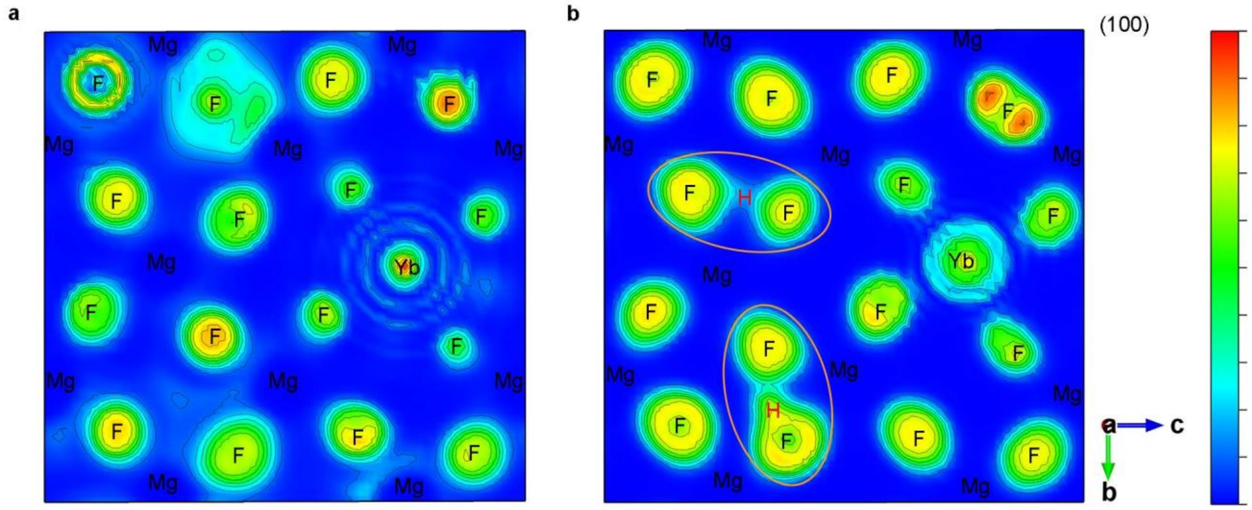

Supplementary Fig. 20 | ELF calculations verified the formation of F−H···F bonds. Electron localization function (ELF) of **a**, $NaMgF_3$:Yb^{3+}_{Mg}-V_{Na} and **b**, $NaMgF_3$:Yb^{3+}_{Mg}-V_{Na}-$3H_i$ in the (100) crystal plane. The formed F−H···F bonds are circled in orange.

ELF calculations showed that interstitially doped H^+ ions form stable F−H···F bonds[13,14] with surrounding F^- ions in $NaMgF_3$:Yb NCs (bond length of 2.3–2.4 Å, marked by orange circles in Supplementary Fig. 20), accompanied by changes in the charge density distribution around Yb^{3+}, which corroborates the occurrence of crystal-field perturbation effects.

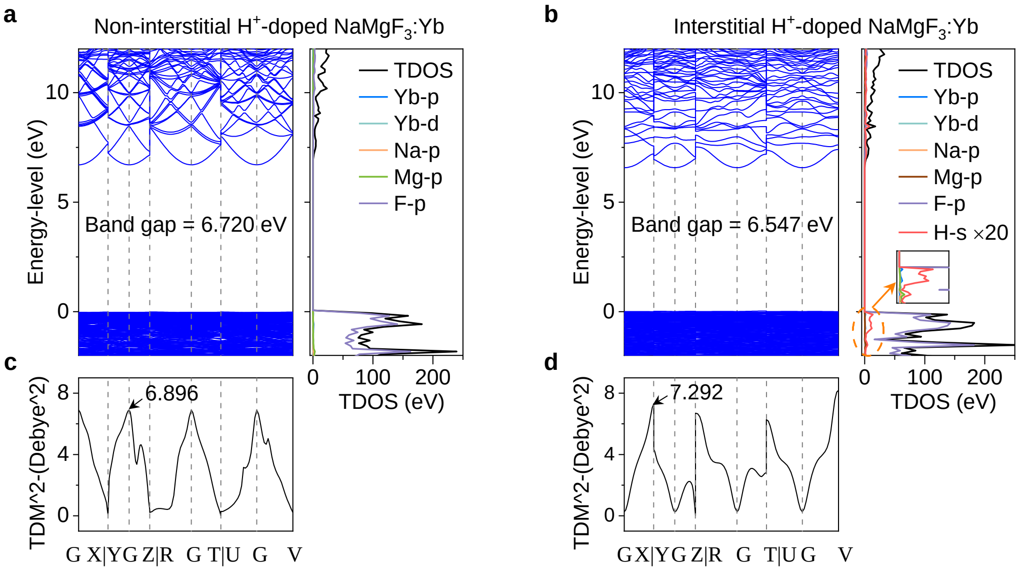

Supplementary Fig. 21 | Effect of interstitial H^+-doping on the band structure and dipole transition matrix elements of $NaMgF_3$:Ln. DFT-calculated electronic band structures and their corresponding projected DOS for **a**, non-interstitially H^+-doped $NaMgF_3$:Yb NCs and **b**, interstitially H^+-doped $NaMgF_3$:Yb NCs. DFT-calculated transition matrix elements for **c**, non-interstitially H^+-doped $NaMgF_3$:Yb NCs and **d**, interstitially H^+-doped $NaMgF_3$:Yb NCs. Source data are provided as a Source Data file.

DFT calculations showed that the introduction of interstitial H^+ ions had a limited effect on the band gap, decreasing it by only 0.173 eV. However, the corresponding density of states (DOS) showed that interstitial H^+ ions contributed to the valence band. This indicates that, although the interstitial H^+ ions had a limited effect on the crystal structure, they still affected the charge distribution within the crystal (Supplementary Figs. 21a and 21b). Additionally, the effect of interstitial H^+ ions on the transition dipole moment can be observed through first-principles DFT calculations. Prior to the introduction of H^+ ions, the dipole transition matrix elements of $NaMgF_3$:Ln were mainly concentrated at the high-symmetry G point (Supplementary Fig. 21c). Therefore, the radiative transition at the $4f^N$ level was parity forbidden. The introduction of interstitial H^+ ions produced an additional field and disturbed the original field of the crystal, resulting in the transfer of the transition dipole moment elements from the high-symmetry G point to the low-symmetry X|Y point (Supplementary Fig. 21d). The existence of dipole transition matrix elements with low

symmetry can promote the mixing of opposite-parity configurations of Ln^{3+} ions, thereby improving the efficiency of the Ln^{3+}-emitter intra-4*f* optical transitions.

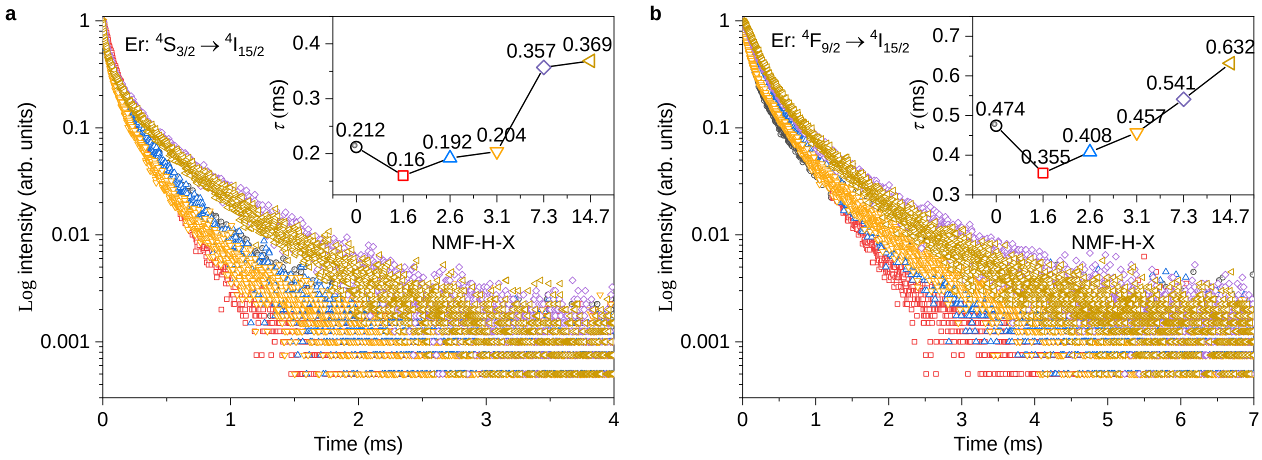

Supplementary Fig. 22 | Effect of interstitial H^+ doping on the Er^{3+} transition rate. Time-resolved decay curves of **a**, $^4S_{3/2} \rightarrow {}^4I_{15/2}$ and **b**, $^4F_{9/2} \rightarrow {}^4I_{15/2}$ transitions of the Er^{3+} emitter in $NaMgF_3$:Yb/Er NCs with different nominal amounts of HAc (X = 0–14.7 mmol HAc) (Na^+ source: NaOH). All the UCL decay curves were measured using a 980 nm pulsed laser at room temperature. The insets show the average UCL lifetimes (τ) from fitting the decay curves with a triple exponential function. Source data are provided as a Source Data file.

The time-resolved decay curves of the $^4S_{3/2} \rightarrow {}^4I_{15/2}$ and $^4F_{9/2} \rightarrow {}^4I_{15/2}$ transitions of the Er^{3+} emitter (Supplementary Fig. 22) show that, as the interstitial H^+ concentration increases, the UCL lifetime first shortens and then prolongs. Comparing NMF-H-0 and NMF-H-3.1, which have a similar particle size, the NMF-H-3.1 NCs have a stronger UCL intensity but shorter UCL lifetime than the NMF-H-0 NCs. This trend was more pronounced at low temperatures (10 K; Fig. 4e). When external conditions such as the size and morphology of the NCs are the same, the influence of the external environment on the nonradiative transition probability W_{NR} can be assumed to be approximately equal. Consequently, according to the relationship $\tau = (A_{ed} + W_{NR})^{-1}$, the shortening of the fluorescence lifetime τ represents an increase in A_{ed}. Therefore, interstitial H^+-doping can simultaneously enhance the radiative transition probabilities of the $^4S_{3/2} \rightarrow {}^4I_{15/2}$ and $^4F_{9/2} \rightarrow {}^4I_{15/2}$ transitions of the Er^{3+} emitter.

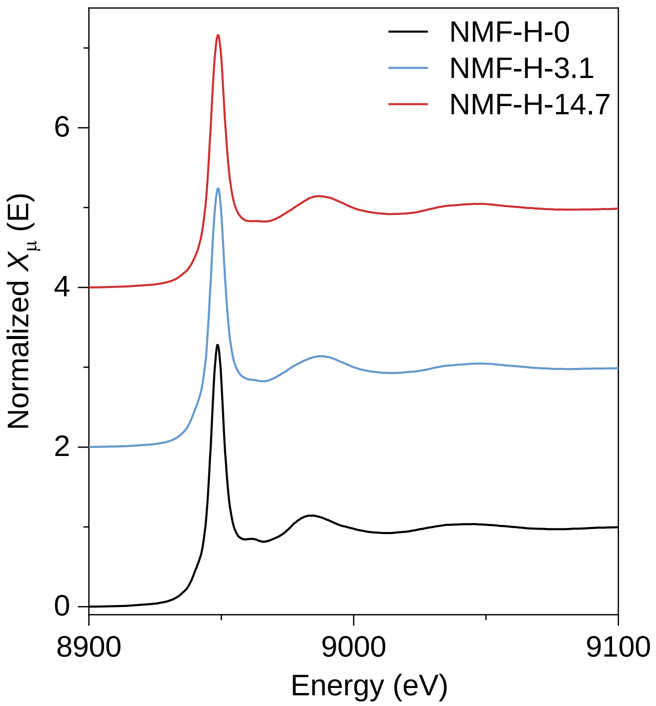

Supplementary Fig. 23 | Yb L_{III}-edge X-ray absorption near edge structure (XANES) spectra for NMF-H-0, NMF-H-3.1, and NMF-H-14.7 NCs. Source data are provided as a Source Data file.

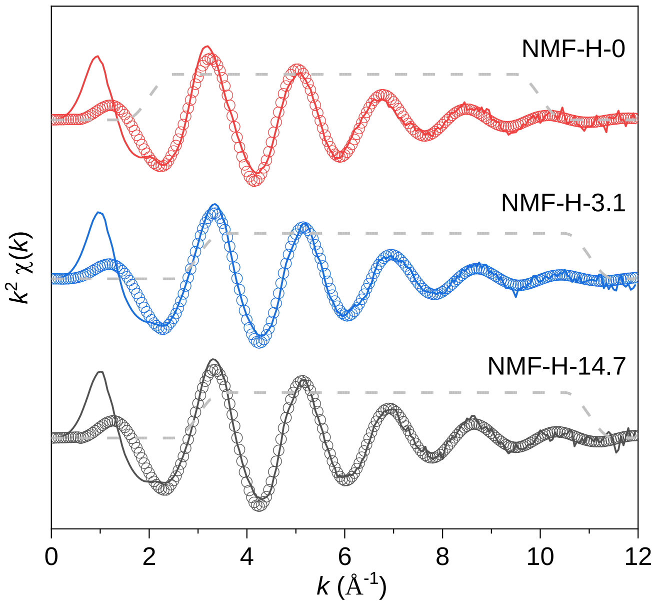

Supplementary Fig. 24 | *k*-space plots (k^2-weighted) of Yb L_{III}-edge EXAFS spectra of NMF-H-0, NMF-H-3.1, and NMF-H-14.7 NCs. Dots represent the experimental data while solid lines show the corresponding fitting curves. The window represents the range of *k*-space involved in curve fitting (3.0–11.0 Å^{-1}). Source data are provided as a Source Data file.

Supplementary Table 6. Coordination number (CN) of Yb^{3+}, average Yb−F interatomic distance (R), Debye−Waller factor (σ^2), and ΔE_0 from curve fitting of the r-space EXAFS spectra[a].

Sample	Shell	CN	R (Å)	σ^2 ($Å^2$)	ΔE_0 (eV)
NMF-H-0	Yb-F	9.4 ± 0.4	2.23	0.012	1.2 ± 0.3
NMF-H-3.1	Yb-F	9.5 ± 0.2	2.18	0.012	0.1 ± 0.2
NMF-H-14.7	Yb-F	9.3 ± 0.2	2.19	0.010	1.4 ± 0.2

[a]The accuracies of the above parameters are estimated as CN, ±20%; R, ±1%; σ^2, ±20%; and ΔE_0, ±20%. The data ranges used for data fitting in k-space (Δk) and R-space (ΔR) were 3.0–11.0 $Å^{-1}$ and 1.0–3.0 Å, respectively.

Supplementary Table 7. Calculated Yb–F bond lengths (Å) and F−Yb−F bond angles (°) of $[YbF_6]^{3-}$ for $NaMgF_3$:Yb NCs with different interstitial H^+ contents, as determined by first-principles DFT calculations.

Parameter	Sample defects			
	$NaMgF_3$:Yb^{3+}_{Mg}-V_{Na}	$NaMgF_3$:Yb^{3+}_{Mg}-V_{Na}-1H_i	$NaMgF_3$:Yb^{3+}_{Mg}-V_{Na}-2H_i	$NaMgF_3$:Yb^{3+}_{Mg}-V_{Na}-3H_i
Crystal structure	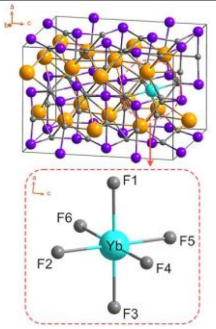	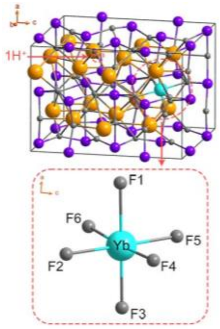	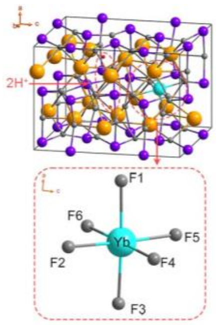	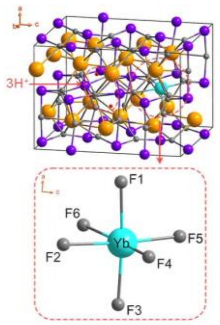
Bond length (Å)				
Yb–F1	2.1209	2.1257	2.1237	2.0984
Yb–F2	2.1174	2.1257	2.1079	2.1538
Yb–F3	2.1188	2.1223	2.1246	2.107
Yb–F4	2.111	2.117	2.1048	2.1017
Yb–F5	2.1058	2.1005	2.0923	2.1019
Yb–F6	2.1154	2.1253	2.1157	2.1436
Bond angle (°)				
F1−Yb−F2	86.71	87.321	85.263	82.856
F1−Yb−F4	90.533	89.258	91.745	87.237
F1−Yb−F5	92.93	91.522	91.497	92.93
F1−Yb−F6	87.697	90.322	88.691	87.419
F3−Yb−F2	92.839	90.087	92.625	91.852
F3−Yb−F4	89.606	89.508	90.018	92.53
F3−Yb−F5	87.52	91.096	90.688	92.364
F3−Yb−F6	92.153	90.787	89.489	91.71
F2−Yb−F4	89.293	88.947	87.802	88.43
F4−Yb−F5	91.161	92.318	89.95	92.293
F5−Yb−F6	90.301	90.425	91.556	99.722
F6−Yb−F2	89.234	88.304	90.722	79.237

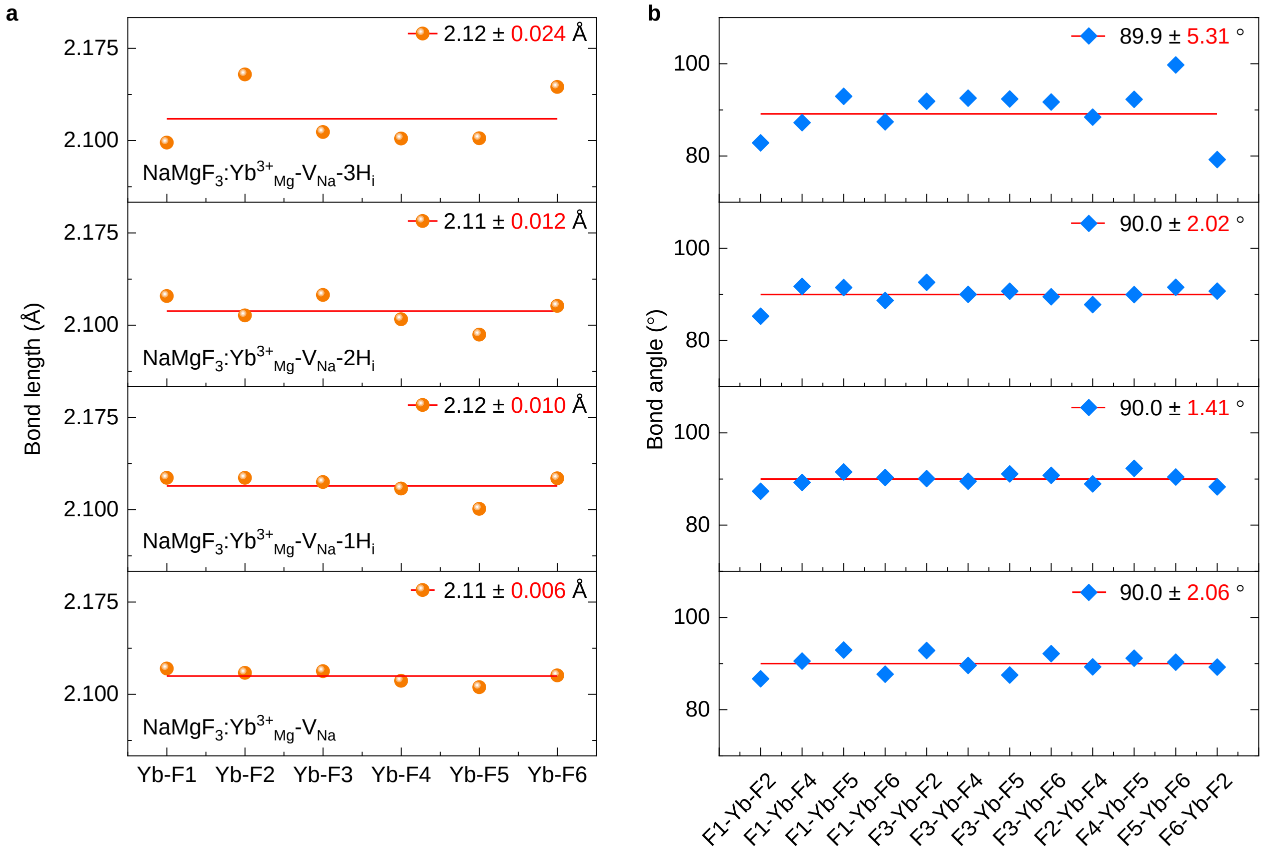

Supplementary Fig. 25 | Effect of interstitial H^+ on sublattice structure of $[LnF_6]^{3-}$. Distribution of **a**, Yb–F bond lengths and **b**, F–Yb–F bond angles within $[YbF_6]^{3-}$ octahedral clusters with different numbers of interstitial H^+ ions (0–$3H_i$), as determined by first-principles DFT calculations. Because Ln^{3+} ions have similar atomic structures, Yb was used as a representative Ln^{3+} ion. The mean ± standard deviation bond lengths and angles are displayed in the upper right corner of each panel. Source data are provided as a Source Data file.

Supplementary Fig. 25 shows that the dispersion of the bond lengths increased only slightly as the number of interstitial H^+ ions increased from zero to two (standard deviation increases by 0.006 Å), indicating that interstitial H^+-doping had a limited effect on the dispersion of bond lengths. The eight Ln–F bonds can therefore be considered of equal length. Conversely, the standard deviation of the bond angles (F–Ln–F) was smaller with one or two interstitial H^+ ions (mean ± standard deviation of 90° ± 1.41° and 90° ± 2.02°, respectively, compared to a standard deviation of 2.06° for undoped H^+). This indicates that when the number of interstitial H^+ ions is one or two, the $[LnF_6]^{3-}$ clusters are

more inclined to form perfect octahedra with high local structural symmetry. Under such high-symmetry conditions, the R/G intensity ratio of Er^{3+} will increase slightly[15]. Therefore, in the experiments, the R/G ratios of samples NMF-H-2.6 and NMF-H-3.1 were slightly higher than those of the samples with higher interstitial H^+ contents (Supplementary Fig. 8). In addition, the limited change in the symmetry of the $[LnF_6]^{3-}$ sublattice structure at low interstitial H^+ concentrations led to similar thermal enhancement of UCL in samples NMF-H-0 and NMF-H-3.1 (Supplementary Fig. 26).

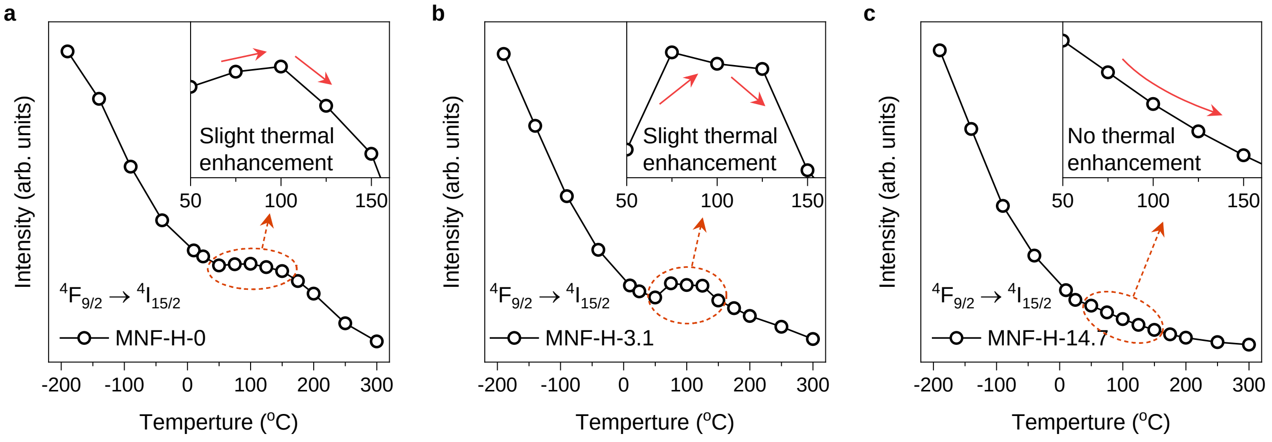

Supplementary Fig. 26 | Temperature-dependence of UCL of interstitially H^+-doped $NaMgF_3$:Yb/Er NCs. Temperature-dependence (−190 to 300 °C) of red UCL intensity ($^4F_{9/2} \rightarrow {}^4I_{15/2}$ transition) of **a**, NMF-H-0, **b**, NMF-H-3.1, and **c**, NMF-H-14.7 NCs (H^+ source: HAc; Na^+ source: NaOH) under 980 nm laser excitation. The insets show an enlargement of the region between 50 and 150 °C. Source data are provided as a Source Data file.

UCL measurements at different temperatures (Supplementary Fig. 26) show that the red UCL intensity generally decreases with increasing temperature. However, both NMF-H-0 and NMF-H-3.1 exhibit slight thermal enhancement of the UCL intensity between 50 and 150 °C (Supplementary Figs. 26a and 26b). This is because the Ln^{3+} (Yb^{3+}/Er^{3+}) dopants occupy the Mg^{2+} lattice sites in $NaMgF_3$, and therefore have a highly symmetrical crystal field environment (S_6 symmetry). The crystal cell undergoes thermal expansion upon increasing the temperature, which reduces the positional symmetry of Ln^{3+} and therefore partially alleviates the parity-forbidden selection rule that is strictly observed in high symmetry environments. Alleviation of the parity-forbidden selection rule facilitates Ln^{3+}-emitter intra-4*f* optical transitions and enhances the PL emission intensity[16]. Both NMF-H-3.1 and NMF-H-0 showed similar thermal enhancement of UCL, indicating that the sublattice structure of Ln^{3+} within them is similar. This further supports the notion that interstitial H^+-doping only causes crystal field perturbation in the crystal field environment around Ln^{3+}, and does not change the corresponding crystal structure. The more pronounced thermal enhancement of UCL for NMF-H-3.1 compared to that for NMF-H-0 is related to the weak UCL intensity of the NMF-H-

0 NCs, which makes the detection of emission signals difficult. Interestingly, the NMF-H-14.7 NCs do not exhibit this UCL thermal enhancement phenomenon (Supplementary Fig. 26c), indicating that extensive interstitial H^+-doping has some effect on the sublattice symmetry.

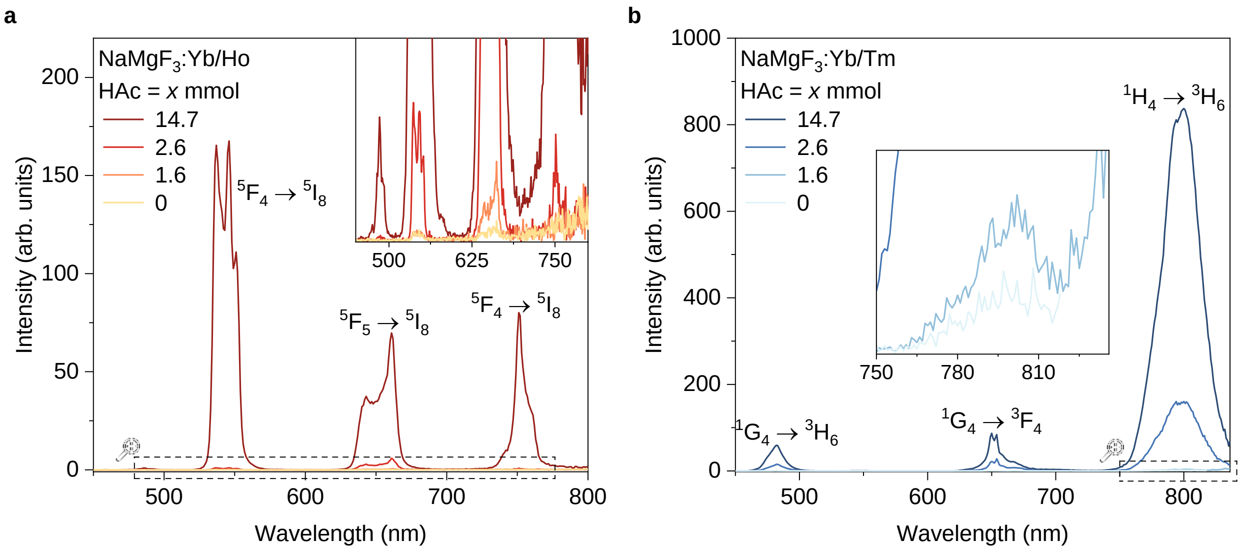

Supplementary Fig. 27 | Generalizability of the interstitial H^+-doping strategy. UCL spectra of **a**, $NaMgF_3$:Yb/Ho (4.5/0.5 mol%) and **b**, $NaMgF_3$:Yb/Tm (4.8/0.2 mol%) NCs interstitially doped with varying H^+ concentrations under 980 nm laser excitation. Owing to the weak luminescence intensity of these samples without interstitial H^+ doping, the inset graphs in **a** and **b** amplify the bands in the 450–800 and 750–836 nm regions, respectively. Source data are provided as a Source Data file.

The UCL spectra of $NaMgF_3$:Yb/Ho (4.5/0.5 mol%) (Supplementary Fig. 27a) contain three characteristic UCL bands of Ho^{3+}, arising from the $^5F_4 \rightarrow {}^5I_8$ (546 nm), $^5F_5 \rightarrow {}^5I_8$ (661 nm), and $^5F_4 \rightarrow {}^5I_8$ (751 nm) transitions, whereas those of $NaMgF_3$:Yb/Tm (4.8/0.2%) (Supplementary Fig. 27b) contain three characteristic UCL bands of Tm^{3+}, arising from the $^1G_4 \rightarrow {}^3H_6$ (481 nm), $^1G_4 \rightarrow {}^3F_4$ (651 nm), and $^1H_4 \rightarrow {}^3H_6$ (800 nm) transitions. All the characteristic UCL bands arising from Ho^{3+} and Tm^{3+} were significantly improved by our designed interstitial H^+-doping strategy, which demonstrates the generalizability of this strategy to different Ln^{3+} ions.

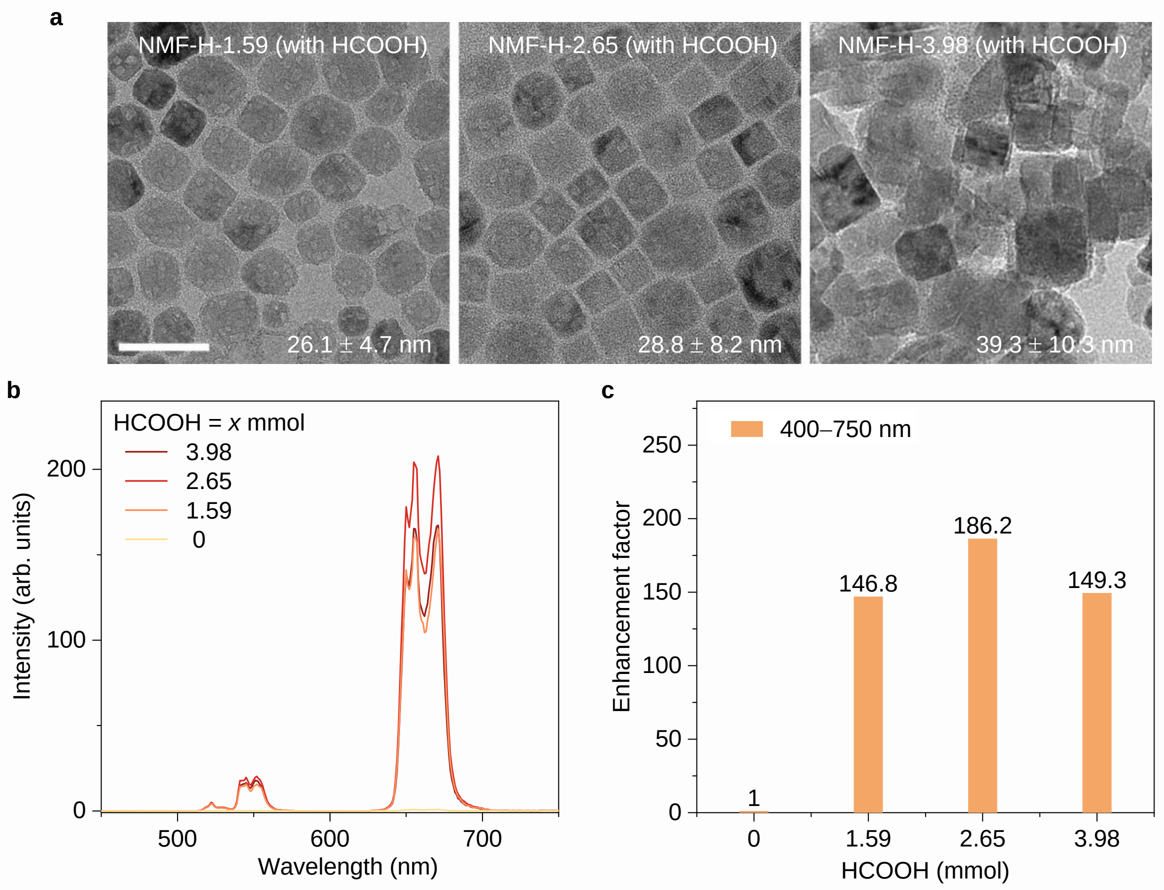

Supplementary Fig. 28 | Effect of formic acid (HCOOH) as a H^+ source on the $NaMgF_3$:Yb/Er NC size, morphology, and UCL intensity. a, TEM images of NMF-H-X (with HCOOH) NCs with X = 1.59, 2.65, and 3.98 mmol nominal HCOOH (Na^+ source: NaOH). The NC size was calculated from the sizes of 100 NCs in typical TEM images, and is given as the mean ± standard deviation at the bottom of each panel in **a**. Scale bars in **a**: 50 nm. **b**, UCL spectra and **c**, corresponding enhancement factors of NMF-H-X (with HCOOH) NCs (nominal amounts of 0–3.98 mmol HCOOH) under 980 nm diode laser excitation with a power density of 50 W/cm^2. The maximum UCL enhancement factor was 186.2 at 2.65 mmol HCOOH. Source data are provided as a Source Data file.

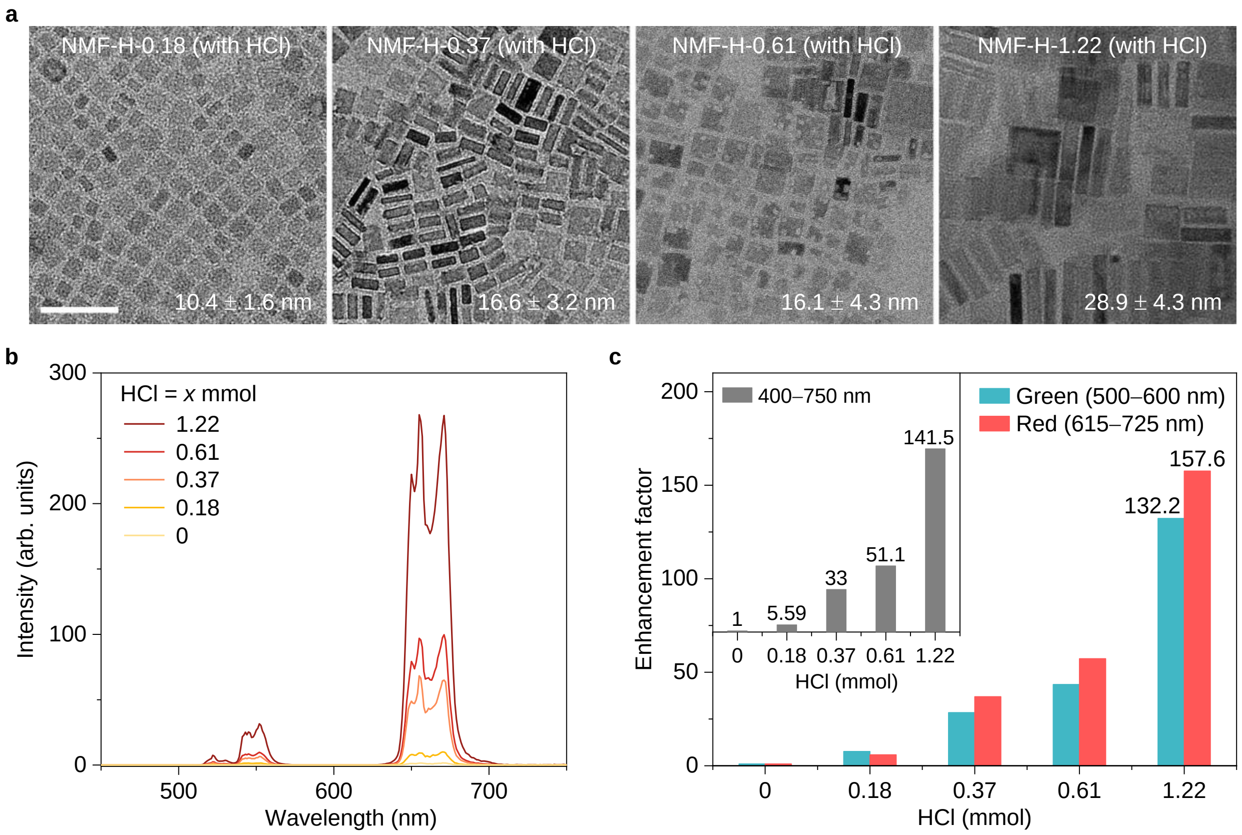

Supplementary Fig. 29 | Effect of HCl as H^+ source on the $NaMgF_3$:Yb/Er NC size, morphology, and UCL intensity. a, TEM images of NMF-H-X (with HCl) NCs with X = 0.18, 0.37, 0.61, and 1.22 mmol nominal HCl (Na^+ source: NaOH). The NC size was calculated from the sizes of 100 NCs in typical TEM images, and was given as the mean ± standard deviation at the bottom of each panel in **a**. Scale bars in **a**: 50 nm. **b**, UCL spectra and **c**, corresponding green ($^2H_{11/2}$,$^4S_{3/2}$ → $^4I_{15/2}$ of Er) and red ($^4F_{9/2}$ → $^4I_{15/2}$ of Er) UCL enhancement factors of NMF-H-X (with HCl) NCs (nominal amounts of 0–1.22 mmol HCl). The inset in **c** shows the enhancement factor of the integrated UCL intensity from 400 to 750 nm. The maximum UCL enhancement factor was 141.5 at 1.22 mmol HCl. Source data are provided as a Source Data file.

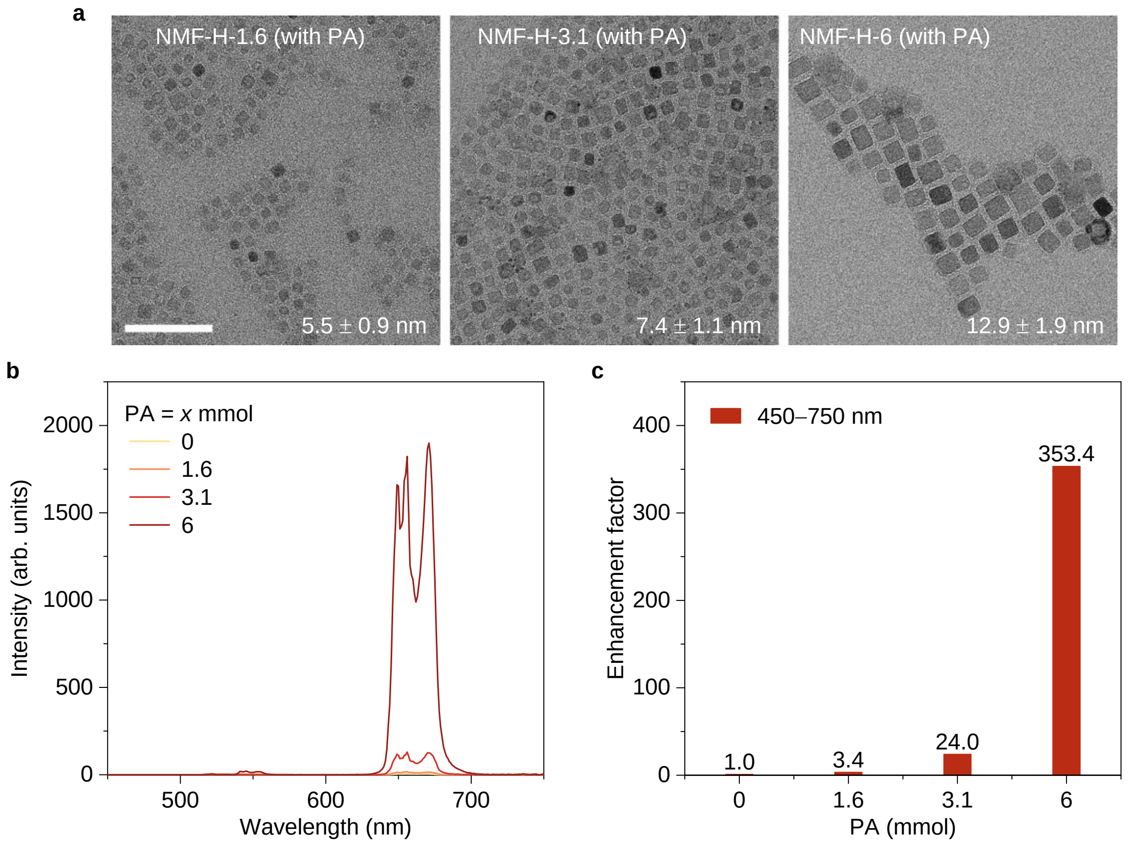

Supplementary Fig. 30 | Effect of propionic acid (PA) as a H^+ source on the $NaMgF_3$:Yb/Er NC size, morphology, and UCL intensity. a, TEM images of NMF-H-X (with PA) NCs with X = 1.6, 3.1, and 6 mmol nominal PA (Na^+ source: NaOH). The NC size was calculated from the sizes of 100 NCs in typical TEM images, and is given as the mean ± standard deviation at the bottom of each panel in **a**. Scale bars in **a**: 50 nm. **b**, UCL spectra and **c**, corresponding enhancement factors of NMF-H-X (with PA) NCs (nominal amounts of 0–6 mmol PA) under 980 nm diode laser excitation with a power density of 50 W/cm^2. The maximum UCL enhancement factor was 353.4 at 6 mmol PA. Source data are provided as a Source Data file.

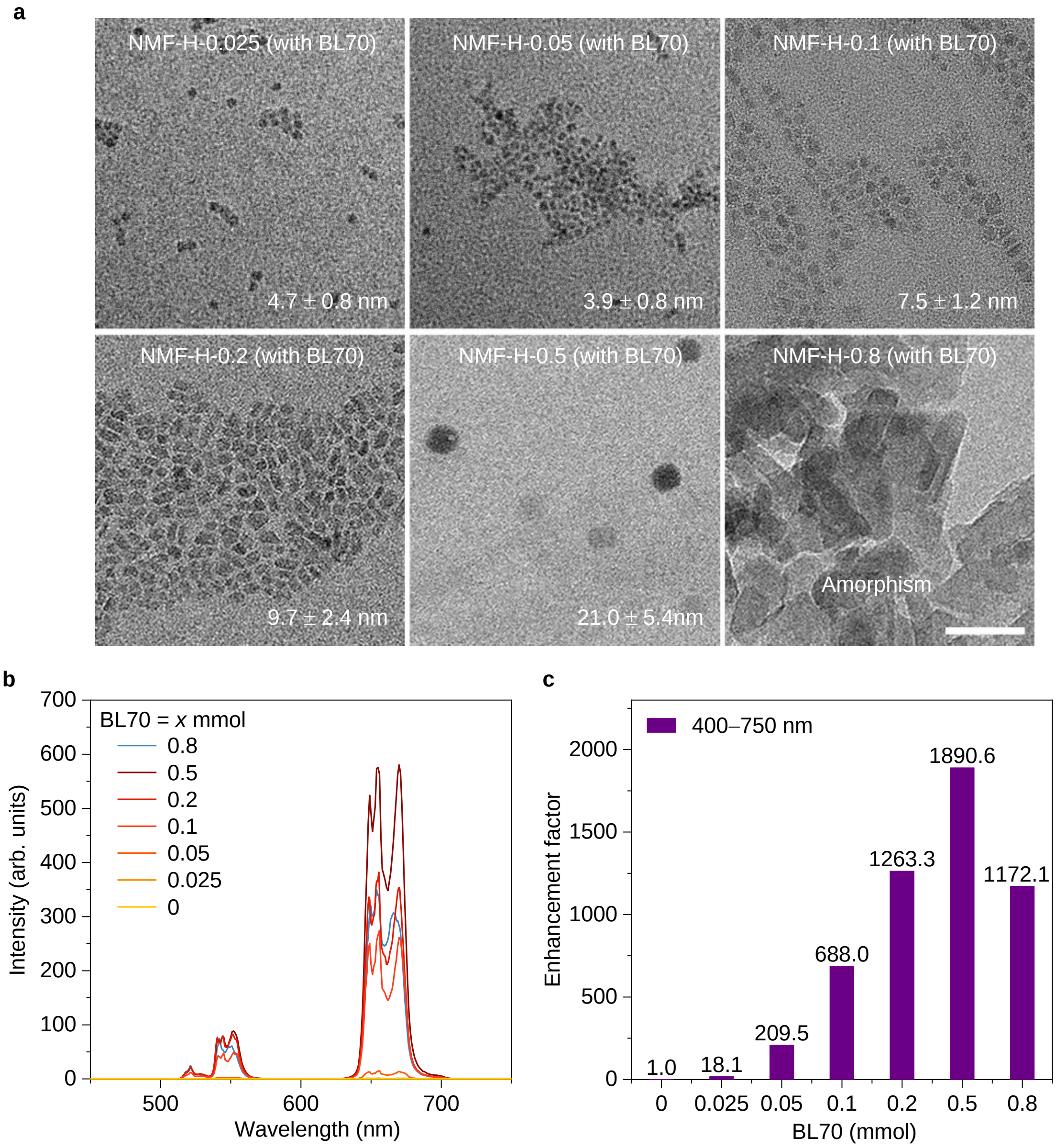

Supplementary Fig. 31 | Effect of BL70 as a H^+ source on the NaMgF$_3$:Yb/Er NC size, morphology, and UCL intensity. **a**, TEM images of NMF-H-X (with BL70) NCs with X = 0.025, 0.05, 0.1, 0.2, 0.5, and 0.8 mmol BL70 (Na^+ source: NaOH). The NC size was calculated from the sizes of 100 NCs in typical TEM images, and is given as the mean ± standard deviation at the bottom of each panel in **a**. Scale bars in **a**: 50 nm. **b**, UCL spectra and **c**, corresponding enhancement factors of NMF-H-X (with BL70) NCs (nominal amounts of 0–0.8 mmol BL70) under 980 nm laser excitation with a power density of 50 W/cm^2. The maximum UCL enhancement factor was 1890.6 at 0.5 mmol BL70. Source data are provided as a Source Data file.

Supplementary Note 1. Effect of different organic acids as H^+ sources

Of the five organic acids used as H^+ sources (HAc (Figs. 2a and 3) and HCOOH, HCl, PA, and BL70 (Supplementary Figs. 28–31, respectively)), BL70 is the strongest organic acid with a relatively high molecular weight. Therefore, BL70 promoted the release of H^+ ions into the $NaMgF_3$:Yb/Er lattice and produced the strongest UCL intensity augmentation effect (1890.6-fold) at a lower nominal addition. This also confirmed that H^+-induced crystal-field perturbation can enhance the UCL intensity with high efficiency.

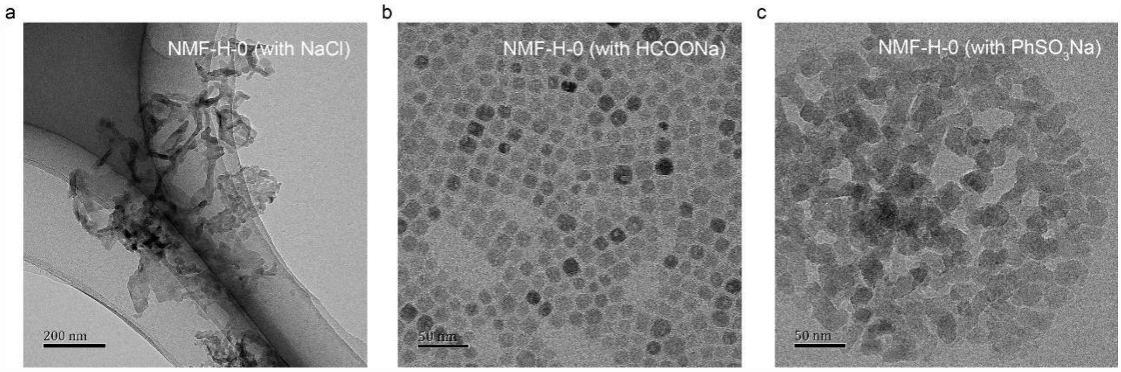

Supplementary Fig. 32 | Effects of different Na^+ sources on the morphology of NMF-H-0 NCs. TEM images of NMF-H-0 NCs synthesized with different Na^+ sources: **a**, NaCl, **b**, HCOONa, and **c**, $PhSO_3Na$. The morphologies of these NCs were all different, indicating that the use of different conjugate bases (Cl^-, $HCOO^-$, and $PhSO_3^-$) results in NCs with different morphologies. Among them, Cl^- and $PhSO_3^-$ have a negative effect on the NC morphology, with the NCs exhibiting undefined shapes and agglomeration.

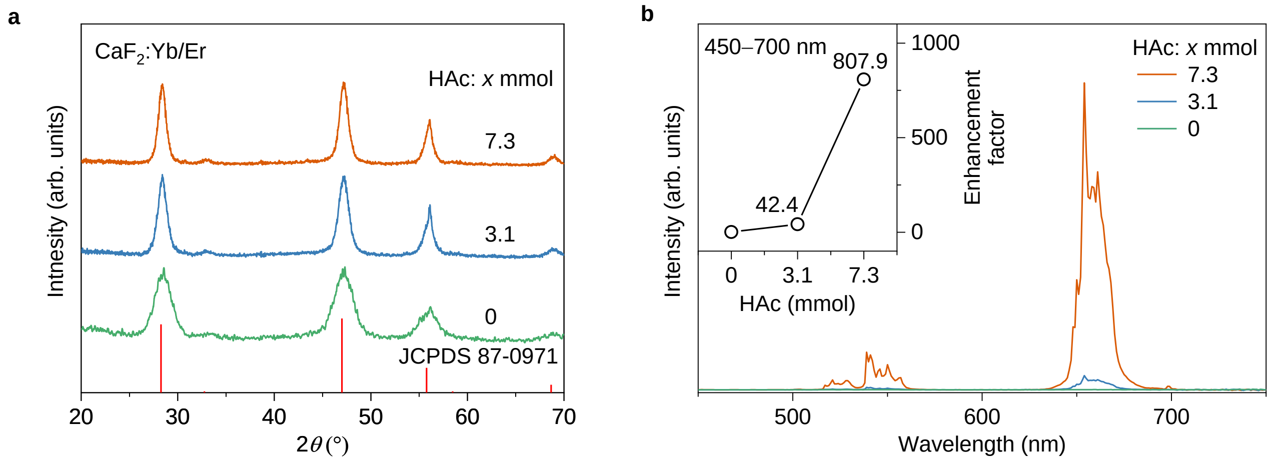

Supplementary Fig. 33 | Effect of interstitial H^+-doping strategy on CaF_2:Yb/Er (Yb/Er = 20/2 mol%) (CaF-H-X) NCs. a, Powder XRD patterns and **b**, typical UCL spectra of CaF-H-X NCs as a function of the nominal amount of HAc used in the synthesis procedure (X = 0–7.3 mmol HAc). The inset in **b** shows the corresponding UCL enhancement factors. All the diffraction peaks matched well with the standard pattern of cubic-phase CaF_2 (JCPDS No. 87-0971), indicating that highly crystalline CaF_2 NCs can be formed with different interstitial H^+-doping concentrations. Moreover, the UCL intensity of CaF_2:Yb/Er increased with the nominal addition of HAc. The maximum UCL enhancement factor was 807.9 at 7.3 mmol HAc. Source data are provided as a Source Data fil

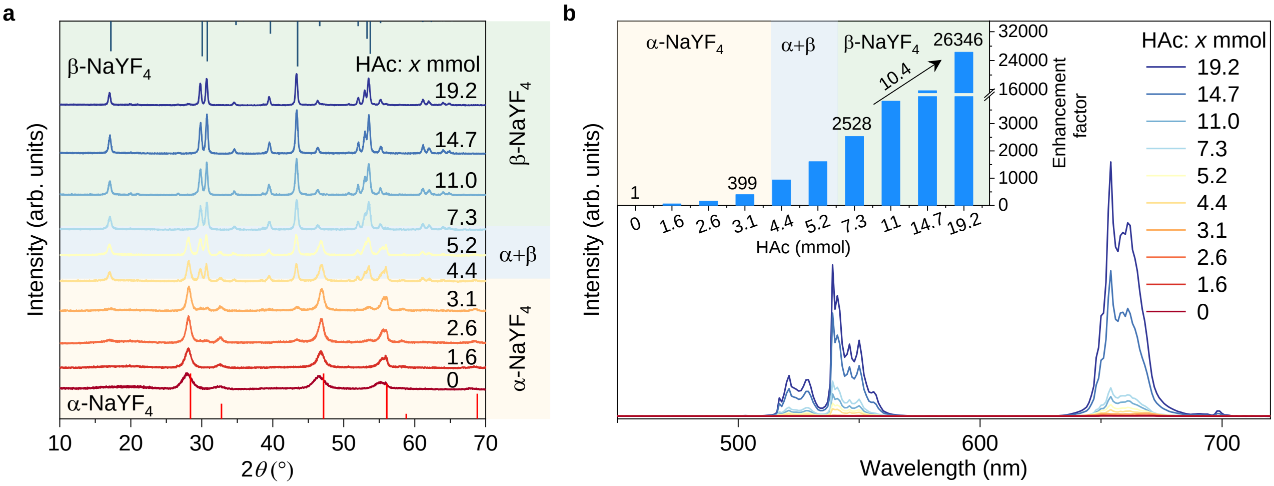

Supplementary Fig. 34 | Effect of interstitial H⁺-doping strategy on NaYF4:Yb/Er (Yb/Er = 20/2 mol%) (NYF-H-X) NCs. a, Powder XRD patterns and **b**, typical UCL spectra of NYF-H-X NCs as a function of the nominal amount of HAc used in the synthesis procedure (X = 0–19.2 mmol HAc). The JCPDS of α-$NaYF_4$ (red vertical line in **a**) and β-$NaYF_4$ (black vertical line in **a**) was No. 06-0342 and 16-0334, respective. The inset in **b** shows the corresponding UCL factors. The maximum UCL enhancement factors were 399 for cubic α-$NaYF_4$ and 10.4 for hexagonal β-$NaYF_4$. Source data are provided as a Source Data file.

Supplementary Table 8. UCQY of CaF-H-X (X = 0, 3.1 and 7.3 mmol HAc) and NYF-H-X (X = 0, 3.1, 7.3 and 19.2 mmol HAc) NCs.

Parameter	CaF-H-X samples			NYF-H-X samples			
	CaF-H-0	CaF-H-3.1	CaF-H-7.3	NYF-H-0	NYF-H-3.1	NYF-H-7.3	NYF-H-19.2
UCQY (%)	0.015	0.122	0.514	0.037	0.160	0.950	2.601

Supplementary Note 2. Application of interstitial H^+-doping strategy to cubic CaF_2, cubic $NaYF_4$, and hexagonal $NaYF_4$:

The interstitial H^+-doping strategy successfully enhanced the UCL intensity of cubic CaF_2 (Supplementary Fig. 33). A UCL intensity enhancement factor of 807.9 was achieved for CaF_2:Yb/Er (20/2 mol%) NCs, while the corresponding UCQY increased from 0.015% to 0.514% (Supplementary Table 8).

The interstitial H^+-doping strategy was also applied to cubic $NaYF_4$ (α-$NaYF_4$) (Supplementary Fig. 34). With nominal HAc additions of less than 3.1 mmol, $NaYF_4$:Yb/Er retained its standard cubic phase (JCPDS No. 06-0342), with a maximum UCL enhancement factor of 399 (UCQY increased from 0.037% to 0.16%). Because the cubic phase α-$NaYF_4$, was not stable at high temperatures, it was easily transformed into a hexagonal phase, β-$NaYF_4$, under external interference at high reaction temperatures. At large interstitial H^+-doping contents (above 3.1 mmol HAc), the cubic-to-hexagonal transition occurs (Supplementary Fig. 34). Nevertheless, when α-$NaYF_4$ was completely transformed to β-$NaYF_4$ (JCPDS No. 16-0334), the interstitial H^+ ions still exerted a crystal-field perturbation effect on β-$NaYF_4$:Yb/Er, enhancing the UCL intensity by a factor of 10.4 (UCQY increased from 0.950% to 2.601%).

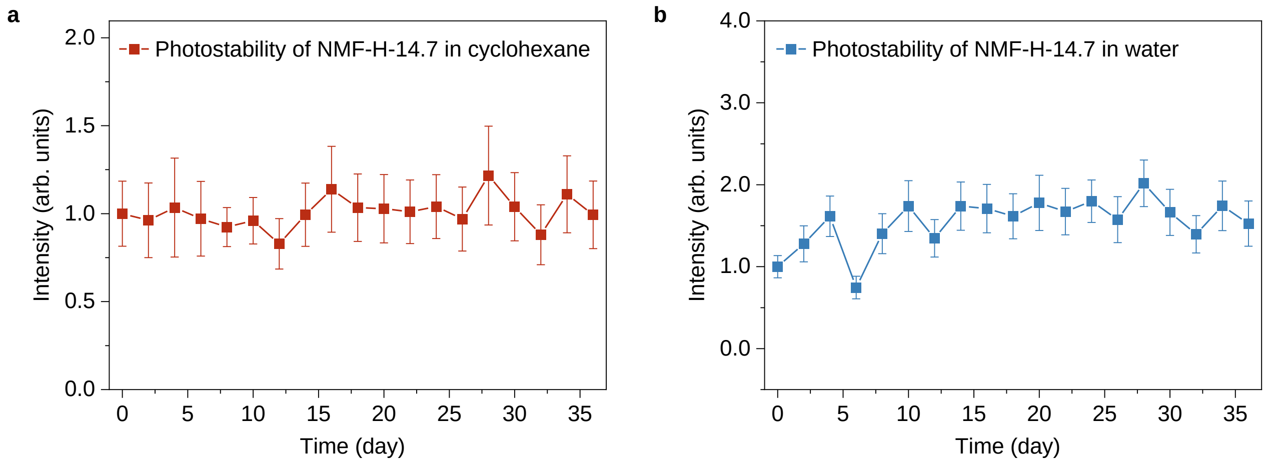

Supplementary Fig. 35 | Long-term photostability of NMF-H-14.7 NCs in **a**, cyclohexane and **b**, deionized water (DSPE-mPEG(2000)-modified). Plots are the mean of three sets of parallel experiments and the error bars represent standard deviation of UCL intensity. The concentration of the solution was 3 mg/mL, and the UCL signal was collected using a continuous 980 nm NIR diode laser with a power density of ~100 W/cm^2 every second day for 36 days. The overall UCL intensity remained almost constant during continuous monitoring over a period of 36 days, strongly suggesting that the as-synthesized NMF-H-14.7 NCs have good stability in cyclohexane as well as in water. The strong electronegativity of F is expected to effectively stabilize the interstitial H^+ ions within the $NaMgF_3$:Yb/Er crystals. Source data are provided as a Source Data file.

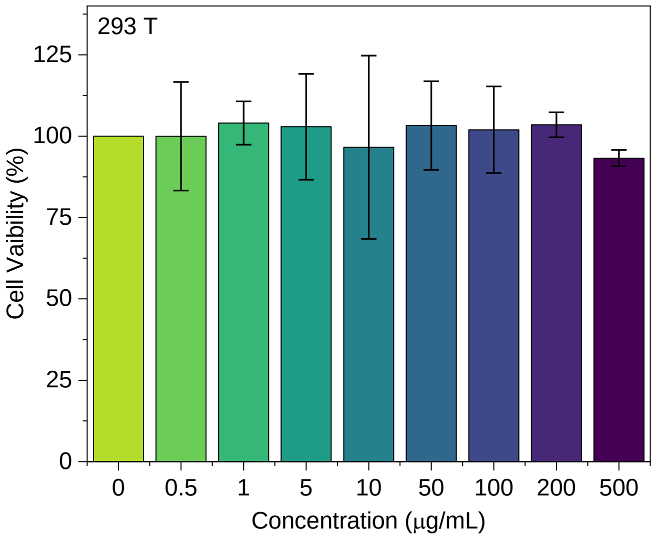

Supplementary Fig. 36 | *In vitro* cytotoxicity of DSPE-PEG(2000)-modified NMF-H-X NCs. Viability of healthy human kidney epithelial (293T) cells after incubation with DSPE-mPEG(2000)-modified NMF-H-14.7 NCs at various concentrations (0, 0.5, 1, 5, 10, 50, 100, 200, and 500 μg/mL) for 48 h. Plots are the mean of three measurements and the error bars represent the standard deviation of cell vaibility. Source data are provided as a Source Data file.

We evaluated the *in vitro* cytotoxicity of DSPE-mPEG(2000)-modified NMF-H-X NCs by using CCK-8 assays on 293T cells. After incubation with modified NMF-H-X NCs at concentrations of 0–500 μg/mL for 48 h, we did not observe any obvious cell viability decrease, indicating the low cytotoxicity of DSPE-mPEG(2000)-modified NMF-H-X NCs for the tested healthy cell lines.

Supplementary References

1. Reid, M. F. & Richardson, F. S. Rationalization of the f-f intensity parameters for transitions between crystal field levels of lanthanide ions. *J. Less-Common Met.* **93**, 113–118 (1983).
2. Souza, A. S., Cortes, G. K., Lima, H. & Couto dos Santos, M. A. The local-field correction factor beyond the Onsager–Böttcher approach: mixing of states from the interaction with atoms in the surrounding medium. *J. Lumin.* **238**, 118292 (2021).
3. Judd, B. R. Optical absorption intensities of rare-earth ions. *Phys. Rev.* **127**, 750–761 (1962).
4. Ofelt, G. S. Intensities of crystal spectra of rare-earth ions. *J. Chem. Phys.* **37**, 511–520 (1962).
5. Yu, C. et al. Influence of Er^{3+} concentration and Ln^{3+} on the Judd–Ofelt parameters in LnOCl (Ln = Y, La, Gd) phosphors. *Phys. Chem. Chem. Phys.* **22**, 7844–7852 (2020).
6. Hao, J., Zhang, Y. & Wei, X. Electric-induced enhancement and modulation of upconversion photoluminescence in epitaxial $BaTiO_3$:Yb/Er thin films. *Angew. Chem. Int. Ed.* **50**, 6876–6880 (2011).
7. Sousa, A., Souza, A. & Lima, H. Defect clustering in an Eu-doped $NaMgF_3$ compound and its influence on luminescent properties. *Mater. Adv.* **2**, 1378–1389 (2021).
8. Wang, J. et al. Phase controllable synthesis of $NaMgF_3$:Yb^{3+}, Er^{3+} nanocrystals with effective red upconversion luminescence. *J. Mater. Sci. Mater. Electron.* **29**, 18320–18330 (2018).
9. Pischedda, V., Ferraris, G. & Raade, G. Single-crystal X-ray diffraction study on neighborite ($NaMgF_3$) from Gjerdingselva, Norway. *J. Mineral. Geochem.* **182**, 23–29 (2005).
10. Yu, L., Li, G., Liu, Y., Jiang, F. & Hong, M. Lanthanide-doped KGd_2F_7 nanocrystals: Controlled synthesis, optical properties, and spectroscopic identification of the optimum core/shell architecture for highly enhanced upconverting luminescence. *Cryst. Growth Des.* **19**, 2340–2349 (2019).
11. Zhong, Y. et al. Boosting the down-shifting luminescence of rare-earth nanocrystals for biological imaging beyond 1500 nm. *Nat. Commun.* **8**, 737 (2017).
12. Hirsh, D. A., Johnson, N. J. J., van Veggel, F. C. J. M. & Schurko, R. W. Local structure of rare-earth fluorides in bulk and core/shell nanocrystalline materials. *Chem. Mater.* **27**, 6495–6507 (2015).
13. Savin, B. A. et al. A new look at electron localization. *Angew. Chem. Int. Ed.* **30**, 409–412 (1991).
14. Pei, Y. et al. Boosting near-infrared luminescence of lanthanide in $Cs_2AgBiCl_6$ double perovskites via breakdown of the local site symmetry. *Angew. Chem. Int. Ed.* **61**, e202205276 (2022).
15. Fu, H. et al. A general strategy for tailoring upconversion luminescence in lanthanide-doped inorganic nanocrystals through local structure engineering. *Nanoscale* **10**, 9353–9359 (2018).
16. Ren, B. et al. Synthesis of core–shell ScF_3 nanoparticles for thermal enhancement of upconversion. *Chem. Mater.* **33**, 158–163 (2021).
